# A newly discovered *Aerococcus urinaeequi* mediates transfer of the pCF10 plasmid via SPI-WT regulation

**DOI:** 10.3389/fmicb.2026.1817926

**Published:** 2026-05-20

**Authors:** Man Zhang, Xiaobo Yang, Rumeng Li, Jingxue Qian, Ruolin Hao, Lin Xu, Qing He, Zhiqiang Shen, Jingfeng Wang, Yu Zhu, Zhigang Qiu

**Affiliations:** 1 The Third Central Clinical College of Tianjin Medical University, Tianjin, China; 2 Military Medical Sciences Academy, Academy of Military Sciences, Tianjin, China; 3 College of Oceanography and Ecological Science, Shanghai Ocean University, Shanghai, China; 4 School of Environmental and Chemical Engineering, Xi’an Polytechnic University, Xi’an, China; 5 Tianjin Key Laboratory of Extracorporeal Life Support for Critical Diseases, Department of Nutrition, Central Hospital, Artificial Cell Engineering Technology Research Center, Tianjin University/Tianjin Third Central Hospital, Tianjin, China; 6 Tianjin Institute of Geriatrics, Tianjin Third Central Hospital Branch, National Medical Quality Control Center of Clinical Nutrition, Tianjin, China

**Keywords:** *Aerococcus urinaeequi*, antibiotic resistance genes, cross-genus transfer, gut microbiota, pCF10 plasmid, signal peptide

## Abstract

**Introduction:**

Pheromone-regulated horizontal transfer serves as the core mechanism for horizontal gene transfer of antibiotic resistance genes, playing a pivotal role in driving the spread of resistance. Given the strict species-specific constraints of this regulatory system, it is imperative to determine whether novel regulatory signal peptides and cross-genus receptors responsive to these signals exist, thereby elucidating its potential for disseminating resistance across broader microbial communities.

**Methods:**

This study isolated and screened a Gram-positive coccus, *Aerococcus urinaeequi* Ae1, from the gut microbiota, and confirmed that Ae1 can undergo intergeneric plasmid transfer with *Enterococcus faecalis* (*E. faecalis*), challenging the conventional understanding that the pCF10 plasmid spreads only within the same species.

**Results and discussion:**

Results showed an intergeneric plasmid transfer frequency of (3.41 ± 0.26) × 10^–3^ in Ae1, which increased to (7.97 ± 1.77) × 10^–3^ upon exogenous addition of the cCF10 signal peptide, indicating cCF10’s regulatory role in this process. Furthermore, the Ae1 signal peptide SPI-WT appeared to functionally resemble the cCF10 mechanism, possibly by acting on the *prgZ/prgX* pathway to promote pCF10 intergeneric transfer. This study suggests that *Aerococcus urinaeequi* can acquire the pCF10 plasmid via intergeneric transfer and provides preliminary evidence that its endogenous signal peptide SPI-WT may play a regulatory role via the *prgZ/prgX* pathway. However, direct proof of natural secretion, physical binding, intracellular uptake, and relief of transcriptional repression is lacking; these remain important questions for future investigation. Nonetheless, our findings provide new insights into the dissemination pathways of intestinal antibiotic resistance genes.

## 1 Introduction

In recent decades, the overuse and misuse of antibiotics have led to the emergence of antibiotic resistance genes (ARGs) and antibiotic-resistant bacteria (ARBs) (Lu et al., 2020; Zhang et al., 2025), posing a significant threat to ecosystems and public health. Globally, at least 700,000 people die annually from diseases related to antibiotic resistance (Minerdi et al., 2023). According to the World Health Organization (WHO), this figure could exceed 10 million by 2050 without appropriate control measures (Ali et al., 2023). Consequently, in-depth research into the transmission mechanisms of ARGs and the development of targeted prevention and control strategies have become urgent priorities in the field of public health.

The emergence and spread of antibiotic resistance in bacteria are primarily driven by DNA mutations, vertical gene transfer (VGT), and horizontal gene transfer (HGT). Among these, HGT is considered one of the primary drivers promoting the development and enrichment of ARGs in the environment (Wang et al., 2019). It encompasses three pathways–conjugation, transformation, and transduction (von Wintersdorff et al., 2016)–typically mediated by mobile genetic elements (MGEs) such as plasmids, bacteriophages, and transposons (Bianco et al., 2022). Among these, conjugation, a primary HGT pathway, refers to the direct physical contact between microorganisms via sex pili, transferring ARGs from donor to recipient bacteria (Ji et al., 2022). Among various MGEs, plasmids are regarded as the primary vectors for ARG dissemination due to their broad host range and efficient autonomous transfer capabilities (Brito, 2021; Wang et al., 2017). Consequently, studying plasmid-mediated conjugative transfer of ARGs holds significant public health implications.

Among the numerous plasmids mediating conjugative transfer, those responsive to bacterial signaling molecules (such as pCF10, pAD1, pAM373, etc.) exhibit particularly noteworthy propagation characteristics (Antiporta and Dunny, 2002; Tao et al., 2022). These plasmids are primarily carried by *E. faecalis*, and their conjugative transfer is regulated by specific pheromones secreted by the bacteria. The transfer frequency is at least 10^3^ times higher than that of ordinary R-plasmids (Inoue et al., 2005; Sterling et al., 2020). This high transfer frequency enables conjugative transfer mediated by such plasmids to rapidly disseminate ARGs, thereby increasing threats to human health and economic development.

The pCF10 plasmid, a well-studied conjugative plasmid in *E. faecalis* (Johnson et al., 2011), has emerged as a model for investigating mobile genetic elements in pheromone-induced conjugative transfer (Dunny et al., 2001). The conjugative transfer it mediates is regulated by two short-peptide pheromones: the positive regulator cCF10 (LVTLVFV) and the negative regulator iCF10 (AITLIFI) (Erickson et al., 2019). Potential pCF10 recipients signal to pCF10-containing donors by releasing cCF10. Upon receiving this signal, donors initiate a series of processes including mutual approach, fusion, and plasmid transfer (Chandler and Dunny, 2008). Meanwhile, iCF10 is primarily secreted by the donor bacterium carrying pCF10 itself. By competitively binding to *PrgX* regulatory protein, it can inhibit the action of cCF10, thereby suppressing plasmid transfer (Kohler et al., 2019). Notably, the tetracycline resistance genes on pCF10 are carried by a conjugative transposon, Tn925. Early *in vitro* studies demonstrated that Tn925 can transfer from *E. faecalis* to Gram-positive bacteria of other genera, such as *Bacillus subtilis*, *Acetobacterium woodii*, and *Leuconostoc oenos*, and integrate into their chromosomes (Christie et al., 1987; Stratz et al., 1990; Zuniga et al., 1996). However, it remains unclear whether the entire pCF10 plasmid can undergo cross-genus conjugative transfer. This characteristic, if confirmed, would not only suggest a broader host range for pCF10, thereby amplifying the risk of environmental dissemination of antibiotic resistance genes (ARGs), but also provide a highly valuable tool for genetic engineering. The present study aims to address this knowledge gap. More critically, it raises a pressing core question: Can pCF10 achieve widespread dissemination beyond *E. faecalis* to other bacterial strains? A positive answer would fundamentally reshape our understanding of the host specificity of pCF10’s pheromone regulatory system, compelling deeper investigation into the molecular mechanisms underpinning its broad dissemination capacity. It would also significantly elevate the risk and evolutionary pace of emerging multidrug-resistant gene complexes in clinical settings.

The gut microbiota constitutes a vital component of the human microbiome, forming a complex ecosystem composed of thousands of microbial species (Fassarella et al., 2021). *E. faecalis*, as common commensal bacteria in the gut, possess the ability to transfer antibiotic resistance genes (ARGs) through conjugative transfer (van den Honert et al., 2020). Previous studies have demonstrated that pCF10-type pheromone-responsive plasmids carried by *E. faecalis* efficiently mediate ARG transfer within the *Enterococcus* genus in the intestinal microenvironment (Hirt et al., 2022; Licht et al., 2002). Even without antibiotic selection pressure, conjugative hybrids formed through conjugation can stably colonize the gut at densities of 10^3^–10^4^ CFU/g feces (Licht et al., 2002). Given the high complexity of the gut microbiota and the potential for pCF10 to undergo cross-genus transfer, we propose a core hypothesis: within the gut microbial community, numerous distinct bacterial species may interact with *E. faecalis*, thereby facilitating the conjugative transfer of the pCF10 plasmid across species. Concurrently, these bacteria themselves may secrete signaling molecules structurally or functionally similar to cCF10, thereby regulating the conjugative transfer process. Confirmation of such cross-genus transfer would hold significant scientific and clinical implications. It would expand the theoretical framework for ARG dissemination, challenge the conventional understanding that “pCF10 transfer is confined to enterococci,” and reveal its ecological risks as an ARG vector. Furthermore, it could guide clinical prevention and control efforts, providing a theoretical basis for designing targeted intervention strategies.

To investigate whether strains other than *E. faecalis* exist within the gut microbiota that can mediate pCF10 plasmid conjugative transfer, this study first performed conjugative transfer using OG1RF (pCF10) as the donor strain with gut microbiota samples. Following conjugation, the system was inoculated onto selective medium containing tetracycline (10 mg/L) for preliminary screening. Recipient bacteria were specifically identified based on the *tetM* resistance gene carried by the pCF10 plasmid. The screened recipient bacteria were then purified and isolated to obtain monoclonal strains. Finally, purified candidate strains were subjected to conjugative transfer validation experiments with OG1RF (pCF10) to confirm their ability to receive the donor pCF10 plasmid. At the same time, streptomycin resistance induction was performed on the isolated recipient bacteria. Conjugates were counted and validated on dual-antibiotic plates using the gradient dilution plating method. Whole-genome sequencing was conducted to analyze the genomic characteristics of the recipient bacteria. Through these experiments, a non-*Enterococcus* Gram-positive bacterium–*Aerococcus urinaeequi*–was successfully isolated from the gut microbiota. This strain serves as a novel recipient for the pCF10 plasmid. This study experimentally demonstrates for the first time that the pCF10 plasmid can be transferred from *E. faecalis* to *Aerococcus urinaeequi*, revealing a previously unknown pathway for the cross-genus transmission of ARGs within the intestinal microenvironment. This discovery not only elucidates the plasticity of pCF10’s host range, but also offers a novel theoretical target for specifically blocking the transmission of resistance genes within complex microbial communities.

## 2 Materials and methods

### 2.1 Collection of laboratory animals and fecal samples

The experiment utilized four standard-grade male Kunming mice (6–8 weeks old, weighing 20–22 g, purchased from Beijing VTLH Laboratory Animal Technology Co., Ltd.). They were housed in a sterile barrier environment maintained at (23 ± 1)°C and (50 ± 5)% humidity, fed standard sterile feed and provided with sterilized drinking water. After 1 week of acclimation, fecal samples were collected. Collection occurred daily between 9:00 and 10:00 AM (the mice’s active defecation period). Feces produced within a 24-h period were collected in clean metabolic cages and transferred to the laboratory for subsequent processing. For sample processing, approximately 200 mg of mouse feces was placed in a 2 mL sterile centrifuge tube. After adding 1 mL of phosphate-buffered saline (PBS) and vortexing, the mixture was centrifuged at 1000 rpm for 30 s at 4°C. The supernatant was decanted, and the pellet was resuspended (by vortexing) and then centrifuged at 1500 rpm for 3 min at 4°C. This process was repeated three times to obtain a fecal bacterial suspension for subsequent use.

### 2.2 Bacteria strains and culture conditions

The conjugation model in this study comprised the *E. faecalis* strain OG1RF (ATCC 47077) carrying the pCF10 plasmid as the donor and the isolated strain Ae1 as the recipient. OG1RF (pCF10) was inoculated into brain heart infusion (BHI) medium (Coolaber Science & Technology, China) containing antibiotic (10 mg/L tetracycline), while the isolated and purified Ae1 were inoculated into antibiotic-free BHI medium. Additionally, Ae1-S was inoculated into BHI medium containing 3 g/L streptomycin and cultured overnight at 37°C and 150 rpm. After centrifugation and washing twice with PBS buffer, the bacterial suspension was resuspended in fresh BHI medium. Specific strains, plasmids, and antibiotic concentrations used are detailed in Supplementary Table 1.

### 2.3 Preliminary screening experiment on plasmid transfer of gut microbiota and *E. faecalis*

Prior to conjugation, the donor strain OG1RF (pCF10) was cultured overnight and collected by centrifugation at 8000 rpm for 3 min at 4°C, wash three times with phosphate-buffered saline (PBS, pH 7.4), then resuspend in BHI and adjust the bacterial suspension concentration to 1 × 10^8^ CFU/ml using OD600. Simultaneously, fecal bacterial suspension was prepared and adjusted to a concentration of 1 × 10^8^ CFU/mL using OD600. Donor bacteria and recipient microbiota were mixed at a 1:1 ratio, incubated at 37°C for 2 h, and sampled. A 0-h conjugation control was also established. To accurately quantify conjugation frequency within the complex gut microbiota, DNA was extracted via boiling method. Real-time quantitative PCR (qPCR) was employed to measure gene abundance of *tetM*, *prgX*, *prgQ*, and *16S rRNA*. The qPCR reaction system (20 μL) comprised: 10 μL PowerUp SYBR Master Mix (A25742, Massachusetts, Applied Biosystems, USA), 2 μL primers, 1 μL DNA, and 7 μL nuclease buffer. *16S rRNA* served as an internal control for calibrating bacterial counts and calculating gene abundance levels. The relative abundance changes of *tetM*, *prgQ*, and *prgX* genes relative to the *16S rRNA* gene represented the efficiency of antibiotic resistance gene conjugation transfer. Details are provided in Supplementary Table 2.

### 2.4 Isolation and purification of strains

Based on preliminary screening results, take 100 μl of fecal bacterial suspension from positive samples and spread them separately onto plates containing *Enterococcus chromogen* agar, *E. coli* chromogen agar, and *Staphylococcus aureus* chromogen agar, each supplemented with 10 mg/L tetracycline. Plates were incubated at 37°C for 24 h to select tetracycline-resistant strains under antibiotic selection pressure. After incubation, plates were retrieved, and target single colonies were picked based on colony color variation. These colonies were then inoculated into corresponding chromogenic media containing 10 mg/L tetracycline for purification. Repeat the streak operation twice to thoroughly eliminate contaminating bacteria. After purification, inoculate the single colonies into BHI liquid medium and incubate at 37°C and 150 rpm for 12–16 h until the logarithmic growth phase. Centrifuge the bacterial suspension at 8000 rpm for 3 min, discard the supernatant, and wash the pellet three times with 7–9 mL of PBS buffer. Add 5 mL BHI medium and 5 mL 40% glycerol to the washed pellet. Mix vigorously to achieve a 1:1 ratio. Aliquot the mixture into labeled sterile Eppendorf tubes and store at −20°C for subsequent experiments.

### 2.5 Plasmid transfer assay between isolated strains and OG1RF (pCF10)

After purification, a single pure colony was obtained and inoculated into BHI liquid medium. It was cultured under shaking conditions at 37°C and 150 rpm for 12–16 h until reaching the logarithmic growth phase, serving as a potential recipient strain. Simultaneously prepare the donor strain: inoculate *Enterococcus faecalis* OG1RF (pCF10) harboring the pCF10 plasmid into BHI liquid medium containing 10 μg/mL tetracycline. Incubate at 37°C with shaking until the logarithmic growth phase is reached. Adjust the bacterial suspension concentration to 1 × 10^8^ CFU/mL to obtain the donor suspension. For the conjugation transfer experiment, mix the two bacterial suspensions at a volume ratio of donor:recipient = 1:1. Incubate the mixture at 37°C in a static incubator for 2 h to facilitate conjugation, then collect samples for detection. A control group was established using samples collected at 0 h (the initial time point of conjugation) to eliminate interference from initial bacterial load on experimental results. Samples were processed using boiling extraction for total DNA. qPCR was employed to detect the relative abundance of the *tetM*, *prgX*, *prgQ* genes, and the internal reference gene *16S rRNA*. The qPCR reaction system is described above.

### 2.6 Mating assay for transfer frequency

As described in Supplementary Text S2, the streptomycin-resistant derivative Ae1-S was induced. The donor strain OG1RF (pCF10) and the recipient strain Ae1-S were inoculated separately into BHI broth containing the appropriate antibiotic concentrations and incubated overnight at 37°C. The cells were washed three times by centrifugation using PBS buffer, then resuspended in BHI broth, and the cell concentrations of both the donor and recipient strains were adjusted to 1 × 10^8^ CFU/mL. The donor and recipient bacterial suspensions were then mixed uniformly in a 1:1 ratio and placed in a 37°C incubator for 2 h of static conjugation. Perform serial dilutions using PBS containing 2 mmol/L EDTA, then spot samples of each dilution onto BHI agar plates containing the corresponding antibiotics. Plates used for zygote screening contained a final concentration of 3 g/L streptomycin and 10 mg/L tetracycline. Concurrently, donor and recipient bacteria were streaked onto plates containing the same concentration of tetracycline or streptomycin, respectively, as counting controls to rule out any mutations. After the bacterial suspension was fully absorbed, the plates were inverted and incubated in a 37°C incubator for 36 h, followed by single-colony counting. Finally, the conjugation transfer frequency was calculated using the following formula:


f=N/TNR

Where N_*T*_ denotes the number of transconjugants and N_*R*_ the number of recipient bacteria.

To minimize counting errors and ensure the precision of the results, each experiment was performed with a minimum of three biological replicates. Moreover, each biological sample was enumerated at least three times.

### 2.7 Verification of the transconjugants

To confirm that the pCF10 plasmid had been transferred to the recipient strain and to assess its stable maintenance, single colonies of the donor [OG1RF (pCF10)], recipient (Ae1-S), and transconjugants were randomly picked from their respective plates. These colonies were then independently passaged for five consecutive generations in their corresponding selective liquid media: the donor in BHI broth containing tetracycline (10 mg/L), the recipient in BHI broth containing streptomycin (3 g/L), and the transconjugants in BHI broth containing both tetracycline (10 mg/L) and streptomycin (3 g/L). Each generation was incubated at 37°C for 8 h (to reach the logarithmic phase) and then transferred at a 1:100 dilution into fresh medium. After the fifth generation, plasmid DNA was extracted from the passaged cells of the donor, recipient, and transconjugants using a plasmid miniprep kit. The extracted plasmids were subjected to agarose gel electrophoresis. Subsequently, PCR amplification was performed using gene-specific primers targeting the pCF10-associated genes prgQ and prgX. All primers were synthesized by Sangon Biotech (Shanghai, China), and their sequences are listed in Supplementary Table 2. The PCR reaction conditions are detailed in Supplementary Table 4. The amplification protocol was as follows: initial denaturation at 95°C for 5 min, followed by 30 cycles of denaturation at 95°C for 10 s, annealing at 60°C for 30 s, and extension at 72°C for 10 s, with a final extension at 72°C for 10 min. PCR products were separated by electrophoresis on a 1% (w/v) agarose gel at 100 V. The gel image was visualized and photographed using a gel imaging system.

### 2.8 Bacterial morphological identification and biochemical tests

Inoculate the glycerol-preserved isolated strain at −20°C into BHI liquid medium and incubate at 37°C with shaking (150 rpm) for 12–16 h until the logarithmic growth phase. Gram staining was performed, and the morphological structures of bacterial cells were observed under a 100× optical microscope with image documentation. A single colony was streaked onto blood agar plates in distinct zones using a sterile inoculating loop. The plates were then incubated at 37°C for 24–48 h. Subsequently, colony characteristics including size, color, edge morphology, and the pattern of hemolysis in the surrounding medium were examined and recorded. Simultaneously, conduct biochemical tests on the isolated strains for arabinose, trehalose, saponins, and V-P, with specific experimental procedures referenced in the appendix Supplementary Text S4.

### 2.9 Antibiotic susceptibility testing

The isolated and purified strain was cultured in BHI liquid medium under static conditions at 37°C overnight. The bacterial suspension was adjusted to a concentration of 10^8^ CFU/mL using sterile saline solution. A 100 μL aliquot of the standardized suspension was evenly spread onto the surface of a BHI agar plate. After complete absorption of the inoculum, antibiotic disks were aseptically placed onto the agar surface using sterile forceps, maintaining a minimum inter-disk spacing of 24 mm and a distance of at least 15 mm from the disk center to the plate edge to avoid overlapping of inhibition zones. The plates were then inverted and incubated at 37°C in darkness for 24 h. Following incubation, the diameter of each inhibition zone (IZD) was measured using a Vernier caliper by taking two perpendicular measurements per zone and calculating the mean value. Bacterial drug susceptibility was interpreted according to the criteria provided in the manufacturer’s instructions for the antibiotic susceptibility test strips. The detailed antibiotic susceptibility results of Ae1 are shown in Supplementary Table 3.

### 2.10 Whole genome sequencing

Whole-genome sequencing of single-colony cultures was performed using paired-end sequencing on the Illumina MiSeq platform (Shanghai Paisenuo Biotechnology). Sequencing results underwent assembly and quality control to obtain complete bacterial genome sequences. By aligning these sequences against the NCBI Nucleotide Sequence Database (NT), species annotation information was obtained, enabling bacterial species identification. The resulting whole-genome sequences were further compared with known bacterial strain sequences in the NCBI database. Phylogenetic trees were constructed using MEGA X software to clarify the evolutionary relationships between the isolated strain and other related strains.

### 2.11 Signal peptide sequence screening, cleavage, and synthesis of conserved functional domain candidate regions

Based on whole-genome sequencing results of the isolated strain, bioinformatics analysis was first conducted to screen for signal peptide sequences, ultimately yielding one secreted protein coding sequence (full length 505 bp) containing a Sec/SPI-type signal peptide. Sequence analysis confirmed that the signal peptide cleavage site for this encoded protein is located between amino acids 29 and 30. In the mature peptide sequence after signal peptide removal, the segment “GDLILVGQQLKVKGE” was identified as a candidate conserved functional domain (designated SPI-WT). prgZ binding site mutant (designated SPI-MutZ, sequence: “GDAIAVGAALKVKGE”) and prgX binding site mutant (designated SPI-MutX, sequence: “GDDIKVSFQLKVKGE”). All peptides were custom synthesized by GenScript Biotech (Jiangsu) Co., Ltd., using solid-phase Fmoc synthesis. Fmoc-protected amino acids served as starting materials, with amino acid bonding to the solid-phase carrier and progressive peptide chain extension mediated by an activator. The resulting peptide chains were cleaved from the solid-phase support using specific cleavage reagents, followed by removal of side-chain protecting groups. Purification was achieved via reverse-phase high-performance liquid chromatography (RP-HPLC) to a purity ≥ 98%. The final synthetic product undergoes molecular weight verification via electrospray ionization mass spectrometry (ESI-MS), ensuring measured molecular weights align with theoretical values for each target peptide. Purified peptides are stored sealed at −20°C for future use.

### 2.12 Molecular docking simulation

To elucidate the molecular interactions between the target peptide (GDLILVGQQLKVKGE) and the *PrgX* and *PrgZ* proteins, computational docking simulations were performed in this study. The initial data for the *PrgX* and *PrgZ* protein structures involved in this study were obtained from the RCSB Protein Data Bank (PDB). The three-dimensional structure of the target peptide was predicted using the PEP-FOLD3 tool. Prior to docking, all molecular structures were preprocessed using AutoDock Tools, including hydrogen atom addition, Gasteiger charge assignment, atom type definition, and saving in PDBQT format. Docking grids (60 × 60 × 60 Å, grid spacing 0.375 Å) were centered on the known conserved binding pockets of *PrgX* and *PrgZ*, respectively. Molecular docking simulations were performed using AutoDock Vina. From the generated docking conformations, the lowest-energy, conformationaly plausible optimal conformations were selected for both protein-peptide pairs. Detailed visualization analysis of these optimal complexes was conducted using PyMOL software to elucidate key intermolecular interactions and binding modes, thereby constructing explicit structural models of the target peptide binding to *PrgX* and *PrgZ*.

### 2.13 Scanning electron microscope (SEM)

Centrifuge the donor, acceptor, and conjugation system at 8000 rpm for 3 min at 4°C. The bacterial pellet was fixed with 2.5% glutaraldehyde for 24 h. After rinsing with PBS, it was dehydrated sequentially with 30%, 50%, 70%, 80%, 90%, 95%, and 100% ethanol (10 min per concentration; 100% ethanol applied twice. Finally, the samples were freeze-dried. The morphological structure of the bacteria was analyzed by a SEM (Sigma300, Zeiss, Germany) (Yang et al., 2023).

### 2.14 RNA extraction and real-time quantitative PCR (qPCR)

The total RNA of donor bacteria, recipient bacteria and conjugative mixtures was extracted using the RNA prep Pure Bacteria Kit (Zhongshitongchuang, Tianjin, China). Subsequently, the extracted RNA was reverse-transcribed to cDNA with the Reverse Transcription Kit (Zhongshitongchuang, Tianjin, China). Furthermore, the DNASTAR software was employed to design primer sequences for qPCR analysis. All samples in the experiments were quantified using a CFX96 qPCR instrument (C1000 Bio-RAD, USA). The qPCR reaction system is the same as above. All specific details are presented in Supplementary Table 2.

### 2.15 Statistical analysis

All data were statistically analyzed using GraphPad Prism 9.5 software. Unpaired *t*-tests were performed for comparisons between two groups, while one-way analysis of variance (ANOVA) was used for comparisons among multiple groups. Statistical significance levels are indicated by the number of asterisks: *P < 0.05, **P < 0.01, ***P < 0.001.

## 3 Results

### 3.1 The gut microbiota can efficiently mediate the horizontal transfer of pCF10 plasmids carrying ARGs

**FIGURE 1:**
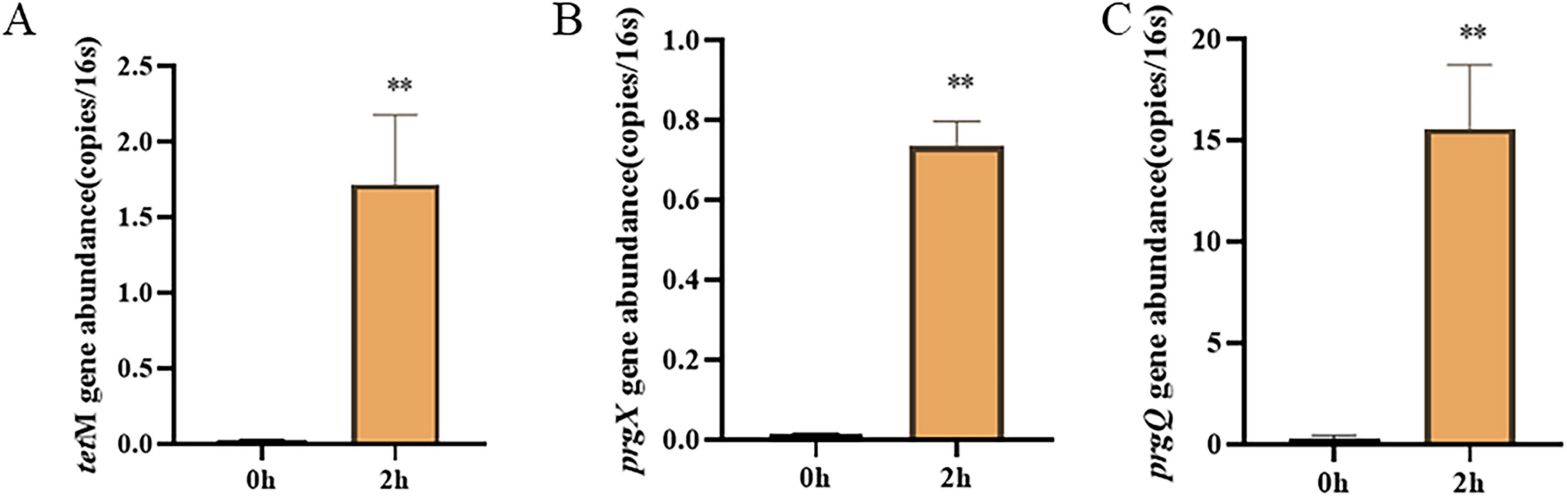
Changes in the abundance of genes representing plasmid transfer in the mouse gut microbiota. **(A)** *tetM*; **(B)** *prgX*; **(C)** *prgQ*. Significant differences were indicated as **P < 0.01.

To validate the transfer capability of gut microbiota in target fecal samples, a model for conjugative transfer of antibiotic resistance genes was established using mouse fecal microbiota (as a representative of gut microbiota) and *E. faecalis* OG1RF (pCF10). Results showed that after a 2-h conjugation transfer experiment, the abundance of the antibiotic resistance gene tetM and the specific genes *prgX* and *prgQ* carried on the plasmid, relative to *16S rRNA* (representing total bacterial load), significantly increased compared to the 0-h conjugation control group (*P* < 0.05) (Figures 1A–C). Specifically, *tetM* gene abundance increased from 0.02 ± 0.008 (copies/16S rRNA) at 0 h to 1.71 ± 0.3 (copies/16S rRNA) at 2 h, representing an 80-fold increase; *prgX* gene abundance rose from 0.012 ± 0.003 (copies/16S rRNA) to 0.7 ± 0.11 (copies/16S rRNA), a 70-fold increase; while *prgQ* gene abundance increased from 0.35 ± 0.05 (copies/16S rRNA) to 12 ± 2 (copies/16S rRNA), representing a 34-fold increase. These findings indicate that the gut microbiota in this fecal sample efficiently mediates horizontal transfer of the pCF10 plasmid, suggesting the presence of strains capable of plasmid transfer with *E. faecalis*. Further analysis revealed highly synergistic changes in the abundance of the three target genes *prgX*, *prgQ*, and *tetM*, closely aligning with the molecular regulatory characteristics of the pCF10 plasmid (Breuer et al., 2018). *prgX* and *prgQ* serve as core regulatory genes for plasmid conjugative transfer, while *tetM* is the tetracycline resistance gene it carries (Singh et al., 2020). Only when *prgX* and *prgQ* initiate conjugation can *tetM* transfer to recipient bacteria along with plasmid DNA (Shu et al., 2013). Consequently, the transfer efficiency of *prgX, prgQ* regulatory genes and *tetM* functional genes is highly synchronized. This finding provides critical data support for subsequent screening of conjugation-competent strains.

### 3.2 A target strain isolated from the gut microbiota capable of undergoing plasmid transfer with OG1RF (pCF10)

**FIGURE 2:**
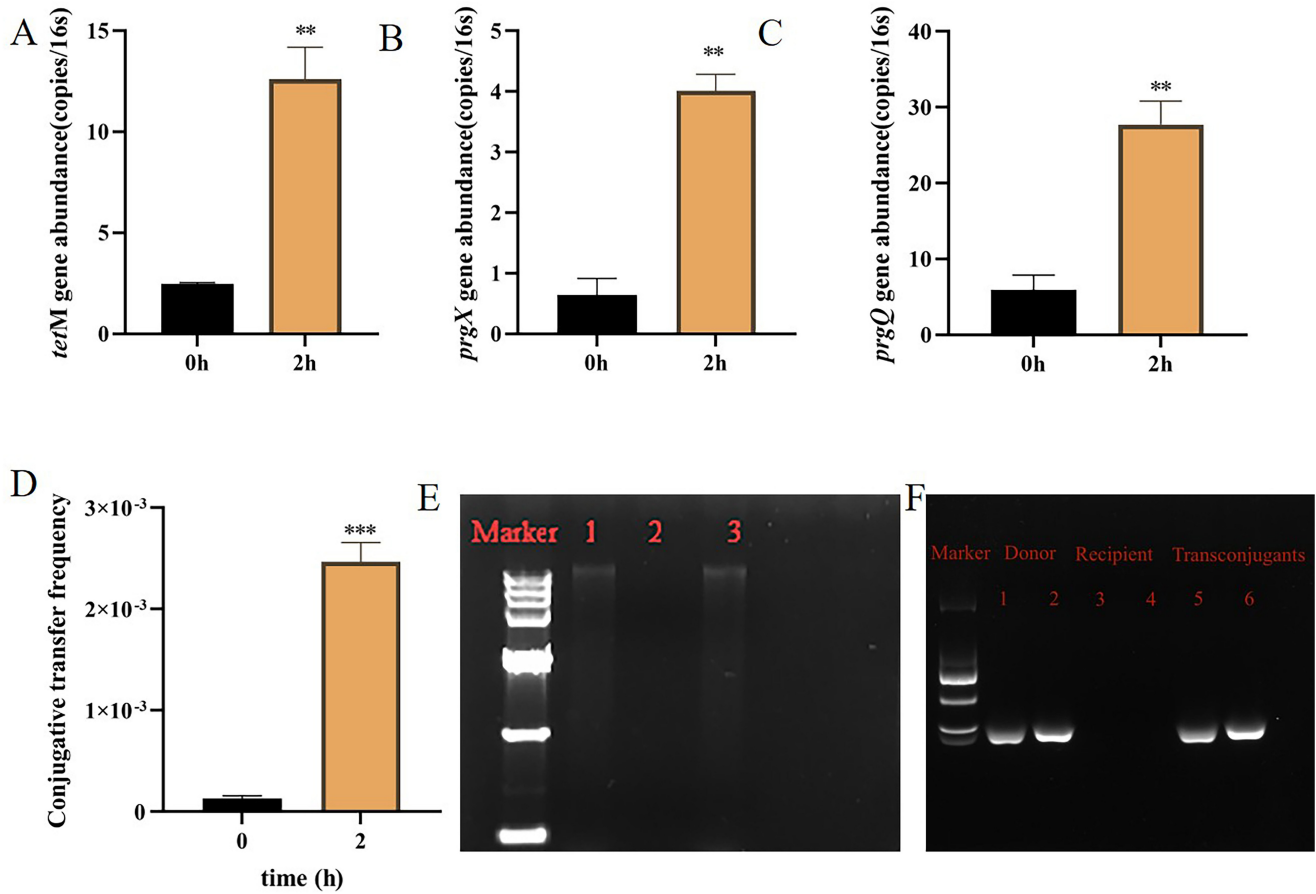
Plasmid transfer experiments between Ae1 and OG1RF (pCF10) changes in the abundance of representative genes **(A)** *tetM*, **(B)** *prgX*, and **(C)** *prgQ* during plasmid transfer between Ae1 and OG1RF (pCF10). **(D)** Transfer frequency assay between Ae1-S and OG1RF (pCF10). **(E)** Agarose gel electrophoresis of plasmids extracted from donor, recipient, and transconjugants after five consecutive generations of passaging. Lane Marker: DNA marker (15000 bp); lane 1: donor strain OG1RF (pCF10); lane 2: recipient strain Ae1-S; lane 3: transconjugants. **(F)** Electrophoresis of PCR products extracted from donor, recipient, and conjugate plasmids. Specific primers targeting *prgX* (125 bp) and *prgQ* (143 bp) were used (see Supplementary Table 2). Marker: DNA ladder (DL2000). Significant differences were indicated as **P < 0.01, ***P < 0.001.

Based on prior confirmation of strains within the mouse gut microbiota capable of conjugative transfer with *E. faecalis*, gut microbiota strains were isolated by streaking on tetracycline selective medium (10 mg/L). Five purified single strains were isolated from the gut microbiota and designated as potential recipient strains. Subsequently, *E. faecalis* OG1RF carrying the pCF10 plasmid (pCF10) was used as the donor strain for plasmid transfer experiments with the five potential recipient strains. Results indicated that only one strain exhibited significantly higher relative *16S rRNA* abundance (representing total bacterial load) for all three target genes compared to the control (*P* < 0.001) (Figures 2A–C). Specifically, *tetM*, *prgX* and *prgQ* gene abundances increased from 3 ± 2 (copies/16S rRNA) at 0 h post-conjugation to 15 ± 2 (copies/16S rRNA) at 2 h, from 1 ± 0.5 (copies/16S rRNA) at 0 h to 5 ± 0.3 (copies/16S rRNA) at 2 h, and from 6 ± 2 (copies/16S rRNA) at 0 h to 40 ± 5 (copies/16S rRNA) at 2 h, respectively–all representing approximately fivefold increases. This indicates the strain’s ability to acquire the pCF10 plasmid via transfer; it was designated Ae1. To further confirm this, the Ae1 genome was extracted for specific PCR analysis. Only the tetM resistance gene was detected, while pCF10-specific genes such as prgQ, prgZ and prgX were not detected, ruling out the possibility of natural carriage of this plasmid (Supplementary Figure 1; see Supplementary Text S1 for detailed methods). To directly verify plasmid transfer and rule out donor proliferation as an alternative explanation, we performed plate spread experiments according to the method described in Supplementary Text S3. OG1RF (pCF10) was co-cultured with Ae1 for 2 h, and control groups consisting of donor-only and recipient-only cells were established. After serial dilutions were plated onto plates containing tetracycline (10 mg/L), no colonies appeared on the recipient plates, donor-only plates produced colonies, but the conjugation system plates yielded a significantly higher number of colonies (Supplementary Figure 3). Quantification at the 10^–5^ dilution showed that the conjugation system produced 7.22 × 10^8^ CFU/mL (mean ± SD, *n* = 3), while the donor-only culture gave only 7.2 × 10^7^ CFU/mL (Supplementary Figure 4). Statistical analysis confirmed that the conjugation system had a significantly higher concentration (*P* < 0.001). Since the recipient alone cannot grow on tetracycline, the extra colonies can only originate from Ae1 that acquired tetracycline resistance via plasmid transfer. This directly demonstrates that the pCF10 plasmid was transferred from OG1RF (pCF10) to Ae1, forming stable transconjugants. Using the streptomycin-resistant recipient Ae1-S, the transfer frequency was (1.28 ± 0.27) × 10^–4^ at 0 h and increased significantly to (2.47 ± 0.19) × 10^–3^ after 2 h of co-culture (Figure 2D, ***P < 0.001). Simultaneously, neither donor nor recipient strains exhibited colony growth when individually plated on dual-antibiotic plates (3 g/L streptomycin + 10 mg/L tetracycline), ruling out false positives; Scanning electron microscopy (SEM) further visualized the direct contact and mating aggregate formation between OG1RF (pCF10) and Ae1 cells during the transfer process, providing direct morphological evidence of cell-to-cell interaction (Supplementary Figure 2). To provide direct structural evidence for the transfer and stable maintenance of the entire pCF10 plasmid, intact plasmids were extracted from OG1RF (pCF10), Ae1-S, and transconjugants and analyzed by agarose gel electrophoresis (Figure 2E). The result showed that the transconjugants harbored a plasmid band of the same size as that of the donor pCF10 plasmid, which was absent in the recipient, confirming that the entire pCF10 plasmid was transferred and is stably maintained as an extrachromosomal element in Ae1. In addition, PCR amplification of plasmid DNA using primers specific for pCF10-related genes (*prgX*, *prgQ*) further confirmed the transfer of the pCF10 plasmid (Figure 2F).

### 3.3 Morphological structure and physiological and biochemical indicator testing of Ae1

**FIGURE 3:**
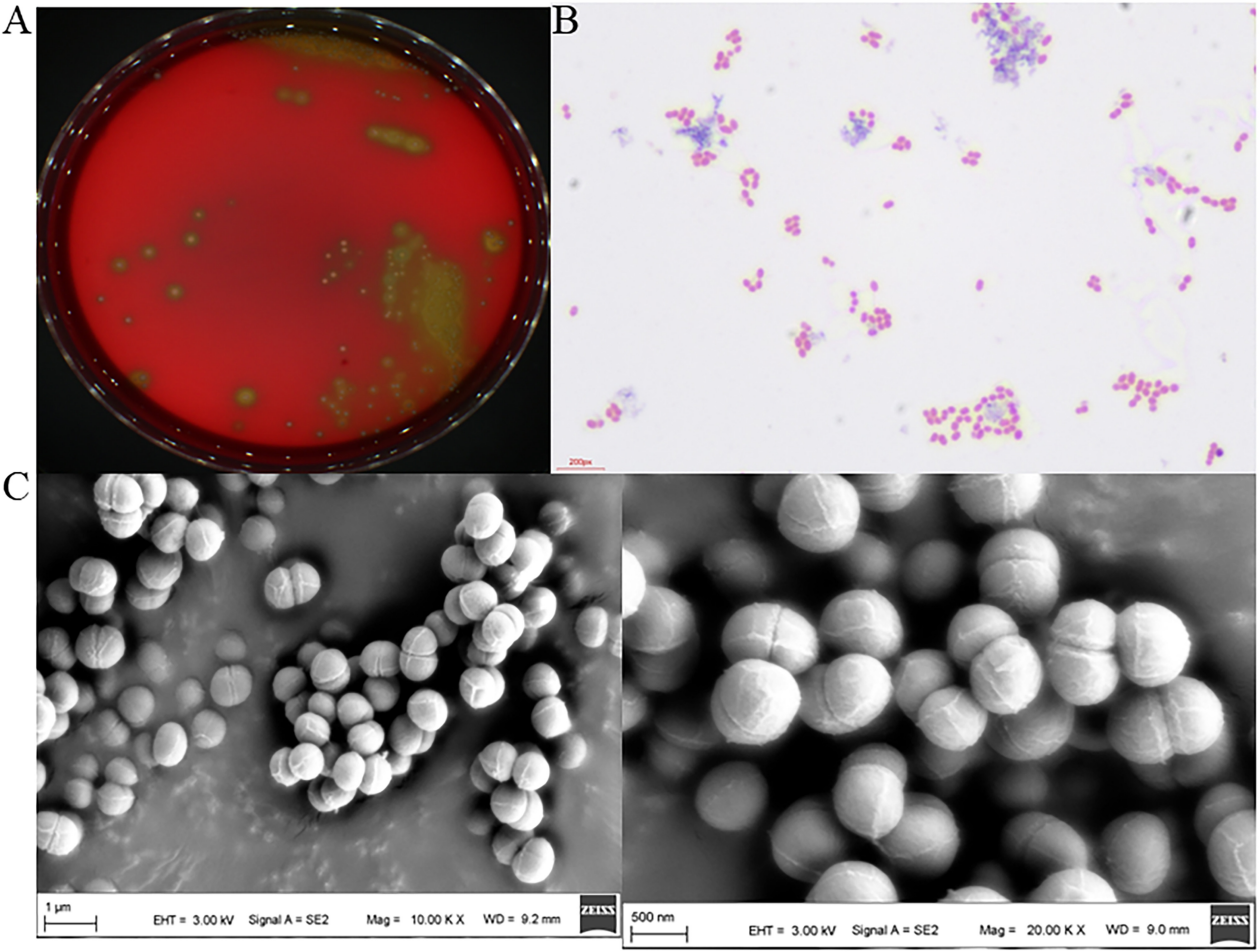
Morphological structure of Ae1. **(A)** Colony morphology of Ae1 on blood agar plates. **(B)** Morphology of Ae1 under optical microscopy. **(C)** Morphological structure of Ae1 under scanning electron microscopy (Left image magnified 10.00 KX, right image magnified 20.00 KX).

To further characterize the species and biological properties of Ae1, the strain was streaked onto Columbia blood agar plates containing 5% defibrinated sheep blood. After incubation at 37°C for 18–24 h, the resulting colonies appeared circular and convex, with smooth surfaces and uniform texture, measuring approximately 0.5–1 mm in diameter, with well-defined edges and a grayish-white translucent appearance. Most colonies were surrounded by a grass-green α-hemolytic zone (Figure 3A). This phenotype closely matched that of *Aerococcus urinae* observed in clinical urine isolates by Zhang et al. (2000). Gram staining revealed Ae1 as Gram-positive cocci predominantly arranged in pairs, tetrads, or clusters, lacking spores or flagella (Figure 3B). This morphology fully corresponds with Christensen et al.’s (2005) observations of 26 strains of *Aerococcus urinae* isolates from diverse sources. Scanning electron microscopy (SEM) further clarified its ultrastructure: cells exhibited typical spherical or ovoid shapes with uniform size (diameter approximately 0.8–1.2 μm), predominantly arranged in pairs, tetrads, or irregular clusters (Figure 3C). These ultrastructural features align with the classic description of *Aerococcus urinae*, providing crucial evidence for preliminary strain identification. Physiological and biochemical tests revealed that Ae1 failed to grow on 6.5% NaCl medium. This negative salt tolerance characteristic aligns with the typical phenotype of *Aerococcus urinae*, enabling clear differentiation from *Enterococcus* species capable of growing in high-salt environments. Additionally, Ae1 exhibited a negative catalase test, with no bubbles formed within 10 s after adding 3% H_2_O_2_ solution. Catalase negativity serves as a key distinguishing indicator between *Aerococcus* and *Staphylococcus* species (Christensen et al., 2005; Facklam and Elliott, 1995). Furthermore, Ae1 can ferment arabinose to produce acid but cannot ferment trehalose. This fermentation profile perfectly matches the metabolic phenotype of *Aerococcus urinae*. Its bile saponin test yielded negative results (no black precipitation zone formed by the binding of saponin and iron ions in the medium). The V-P test also yielded negative results, indicating the absence of the metabolic pathway for acetylmethylmethanol production (Christensen et al., 2005). This finding strongly aligns with the metabolic characteristics of *Aerococcus urinae* (Table 1). In summary, the physiological and biochemical features of Ae1 correspond to the phenotypic characteristics of the clinically isolated *Aerococcus urinae* variant strain. Combined with prior morphological observations, this further confirms the strain as *Aerococcus urinaeequi*.

**TABLE 1:** Biochemical characteristics of Ae1.

Phenotypic characteristics	Test results
Cholestyramine	−
V-P experiment	−
6.5% NaCl growth	−
Enzyme-activated experiment	−
Arabinose	+
Trehalose	−

### 3.4 Whole genome sequencing of isolate Ae1

**FIGURE 4:**
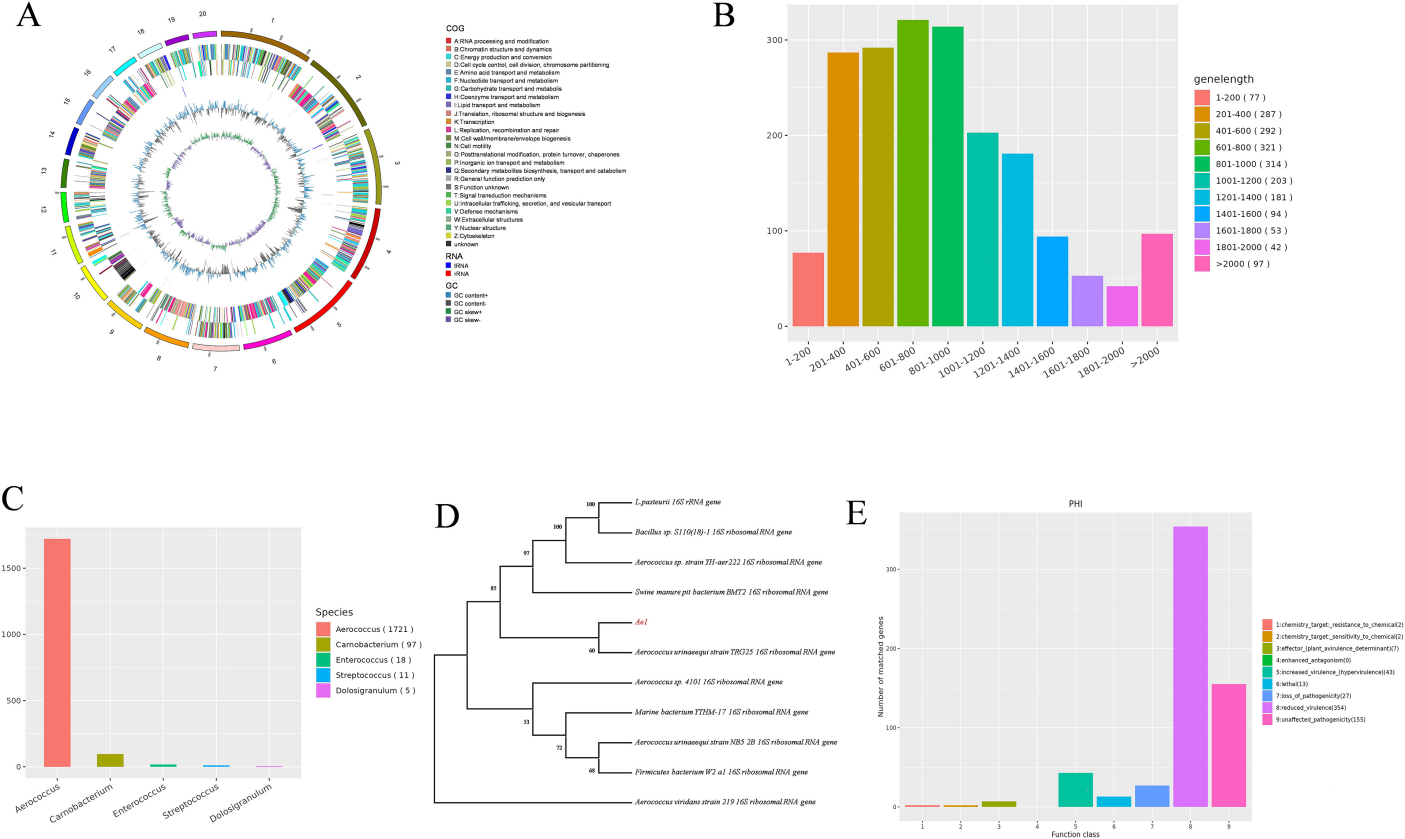
Genomic sequencing characteristics of *Aerococcus urinaeequi* Ae1 strain. **(A)** Circos plot of genome A; **(B)** Bar chart of gene length distribution; **(C)** Species annotation count statistics from NR database; **(D)** Phylogenetic tree based on *16S rRNA* gene sequences; **(E)** Functional classification diagram of PHI genes. **(A–C,E)** adapted from the bacterial genome framework project completion report of Shanghai Paisenuo Biotechnology Co., Ltd. (Project No.: PN20241120064); **(D)** generated based on completion report data and independent analysis results.

Genome sequencing and assembly of Ae1 revealed sequence lengths ranging from 591 to 156,465 bp with a G+C content of 39.4%. Using the Circos circular visualization system, the core features of this strain’s genome were systematically presented: from outer to inner layers, these include the chromosomal karyotype, CDSs on both positive and negative strands (different colors indicating distinct COG classifications of CDSs), tRNAs and rRNAs, GC content, and the innermost layer showing GC skew. This provides an intuitive visualization of the structural and functional relationships within the genome. Gene prediction using GeneMarkS software identified 1,961 genes, with an average length of 905.67 bp, accounting for 85.26% of the total genome sequence. Based on gene length distribution, the predominant ranges were 601–800 bp (321 genes) and 801–1000 bp (314 genes), with the longest ORF reaching 5811 bp. This distribution aligns with typical bacterial gene length patterns, providing foundational data for subsequent functional annotation and structural analysis (Figures 4A,B). Species annotation from the NR database serves as a basis for taxonomic classification. Based on NR database species annotation results, a total of 1,852 protein-coding genes were annotated, with 1,721 genes annotated as belonging to the genus *Aerococcus*, representing the most abundant annotation. This preliminary finding suggests that the strain may belong to the genus *Aerococcus* (Figure 4C). Further analysis using 120 conserved single-copy genes across bacteria, leveraging the GTDB database and GTDBtk classification tool, revealed that Ae1 exhibited the highest average nucleotide identity (ANI) of 97.74% with *Aerococcus urinaeequi* GCF_001543205.1. This ultimately identified the isolate as *Aerococcus urinaeequi*. To validate the classification, specific sequences from Ae1 were compared against NCBI database sequences. The top ten sequences with 100% coverage and >99% similarity were selected for homology analysis. After alignment using the ClustalW tool in MEGAX software, a phylogenetic tree was constructed via the Neighbor-Joining method (Figure 4D). Results indicated that the 16S rRNA sequence of Ae1 showed the highest homology with the standard strain TRG25 of *Aerococcus urinaeequi*, suggesting a strong genetic relationship between them.

Furthermore, alignment with the VFDB database identified five categories of virulence-associated genes in the Ae1 genome: immune regulation-related genes (*ugd*, *DegT*, *hasC*, *gndA*, *galE*, *cps4I*, *wbtP*), stress response protein-related genes (*clpP*, *katA*), adhesion-related genes (*groEL*, *tuf*, *dnaK*), extracellular enzyme-related genes (eno), and regulatory-related genes (*lisR*, *sigA*). Simultaneously, analysis of its resistance genes using the CARD database revealed that Ae1 exhibits resistance to fluoroquinolones, peptides, macrolides, and rifamycin antibiotics. The resistance genes *arlR*, *efrA*, and *efrB* were detected, with the resistance mechanism being antibiotic efflux. Resistance to diaminopyrimidines, peptides, and rifamycin antibiotics was detected with the *dfrE* and *Bsub_rpoB_RIF* genes, indicating antibiotic target substitution as the resistance mechanism; resistant to tetracycline antibiotics, with the *rpsJ* gene (a tetracycline resistance protein identified in *Neisseria gonorrhoeae*) detected; the resistance mechanism involves antibiotic target site mutation. Additionally, numerous genes related to antibiotic target site modification were present, such as *vanR_in_vanF_cl*, *Saur_cls_DAP*, and *Saur_rpoC_DAP*. Genes involved in multidrug resistance mechanisms include *Efac_liaR_DAP* (efflux and target site modification) and *Bsub_rpoB_RIF* (target site modification and target site substitution). The combination of the rpsJ gene with the *efrA/efrB* genes may confer simultaneous resistance to tetracyclines and macrolides in Ae1; The synergistic interaction between the *Bsub_rpoB_RIF* gene (rifamycin target substitution) and the *Saur_rpoC_DAP* gene (rifamycin target modification) could contribute to its resistance to rifamycin antibiotics. Additionally, PHI annotations reveal that Ae1 harbors 354 virulence-reduction-associated genes, potentially altering pathogen metabolic pathways and drug targets. Some virulence-reduced bacteria may increase uptake of certain antibiotics, enhancing therapeutic efficacy; others may reduce antibiotic penetration efficiency through mechanisms like altered cell membrane permeability, complicating treatment (Figure 4E). Furthermore, pathogens harboring virulence-reduced genes that persist long-term within the host may facilitate the spread of drug resistance genes. The findings revealed by whole-genome sequencing–including species classification, virulence, and drug resistance characteristics–are highly consistent with previous morphological observations and physiological-biochemical identification data, further validating the accuracy of strain identification and functional analysis.

### 3.5 The transfer between Ae1 and OG1RF (pCF10) could be regulated by the pheromones cCF10 and iCF10

**FIGURE 5:**
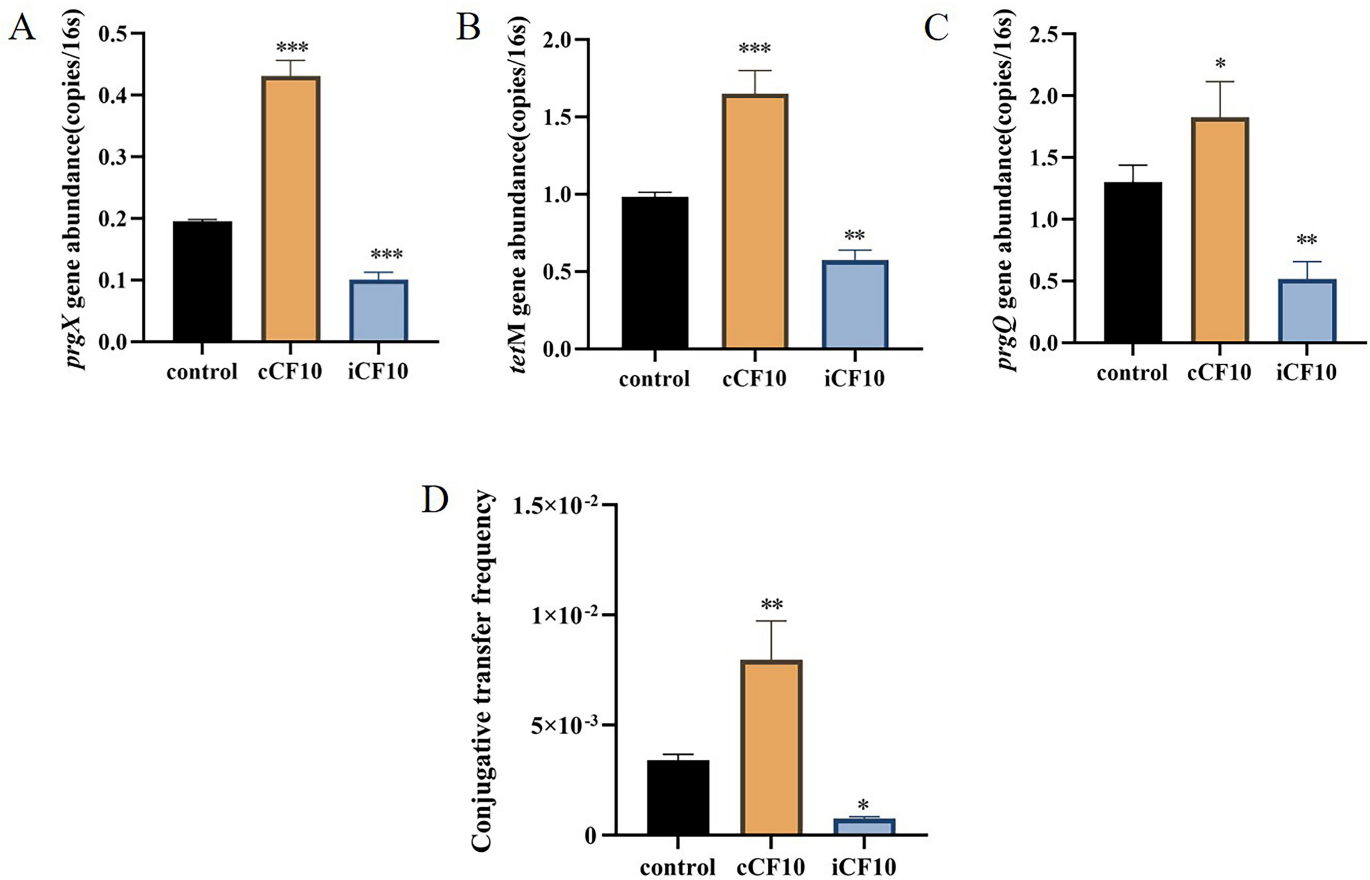
Patterns of ARG gene abundance changes during plasmid transfer mediated by the pCF10 plasmid in cCF10 and iCF10. **(A)** *tetM*; **(B)** *prgX*; **(C)** *prgQ*; **(D)** Plasmid transfer experiment between Ae1-S and OG1RF (pCF10). Significant differences are indicated by *P < 0.05, **P < 0.01, ***P < 0.001.

cCF10 (LVTLVFV) and iCF10 (AITLIFI) are core regulatory molecules mediating bacterial conjugative transfer via the pCF10 plasmid in *E. faecalis* (Chen et al., 2017). They exert opposing roles within the pCF10 plasmid’s transfer regulatory system: cCF10 acts as a positive signal driving the conjugation process, while iCF10 acts as a negative signal to inhibit it, jointly maintaining the precision and ecological adaptability of conjugative transfer. This regulatory model has been thoroughly validated within the *E. faecalis* species. Our laboratory’s prior research on intraspecific conjugative transfer in *E. faecalis* also demonstrated that the pheromone cCF10 significantly increases conjugative transfer frequency, whereas iCF10 inhibits the conjugation process (Yang et al., 2023). Building upon this foundation, our systematic experiments further suggest that this pheromone-regulated intergeneric transfer also occurs between Ae1 and *E. faecalis* OG1RF (pCF10). We have precisely characterized their regulatory effects on this process and identified key concentration thresholds. Using conjugation-related genes (*tetM*, *prgX*, *prgQ*) on the pCF10 plasmid and bacterial *16S rRNA* genes as detection targets, real-time quantitative PCR analysis of gene abundance changes revealed that exogenous addition of 20 ng/ml cCF10 further increased gene abundance by 1.4- to 2.2-fold, consistent with cCF10’s role in promoting conjugative transfer within *E. faecalis*. Conversely, adding 10 ng/ml of the inhibitory pheromone iCF10 reduced abundance by 1.7- to 2.5-fold (*p* < 0.01) (Figures 5A–C). To directly assess the functional effects of cCF10 and iCF10 on conjugative transfer efficiency, a mating assay was performed using the streptomycin-resistant recipient Ae1-S. The transfer frequency was determined by plating serial dilutions on dual-antibiotic plates (streptomycin + tetracycline). As shown in Figure 5D, the natural conjugation frequency was (3.41 ± 0.26) × 10^–3^ (mean ± SD, *n* = 3). Addition of 20 ng/mL cCF10 significantly increased the frequency to (7.97 ± 1.77) × 10^–3^ (*P* < 0.01), whereas treatment with 10 ng/mL iCF10 markedly reduced it to (0.76 ± 0.08) × 10^–3^ (*P* < 0.05). This antagonistic effect aligns with the mechanism of iCF10 within *E. faecalis*, suggesting that this transfer process is positively regulated by cCF10 and negatively inhibited by iCF10.

### 3.6 Ae1 newly identified signal peptide SPI-WT regulates *prgX*, *prgZ* activation for cross-genus transfer

Since the cross-genus transfer from OG1RF (pCF10) to Ae1 is positively regulated by cCF10, we hypothesize that homologous sequences of cCF10 may exist within the Ae1 genome. However, BLASTn alignment results revealed no significant homology between the entire Ae1 genome and the cCF10 gene sequence (matching score < 30%, *E*-value > 1e-5), indicating that Ae1 does not carry cCF10-related coding sequences. This suggests that Ae1 may participate in transfer regulation through its own unique endogenous signaling molecules.

Based on this hypothesis, this study combined SignalP 6.0 prediction results to screen one conserved functional domain fragment containing a Sec/SPI-type signal peptide (SPI-WT) from Ae1 genome-predicted secreted protein sequences. This peptide was obtained via solid-phase peptide synthesis (purity ≥ 95%, with molecular weight verified by MALDI-TOF MS matching the theoretical value). To validate SPI-WT’s regulatory function, different concentrations of SPI-WT (0.1, 1, 10, 20, 100, 1000 ng/ml) were added to conjugation systems. An equal volume of DMSO served as the control. Changes in the abundance of conjugation-related genes (*tetM*, *prgX*, *prgQ*) were detected via qPCR. Results indicated a significant concentration-dependent promotion of transfer by SPI-WT: gene abundance initially decreased then increased with rising peptide concentration, with the 0.1 ng/ml group showing increases from 1.49-fold to 1.92-fold compared to the control (Supplementary Figure 5).

**FIGURE 6:**
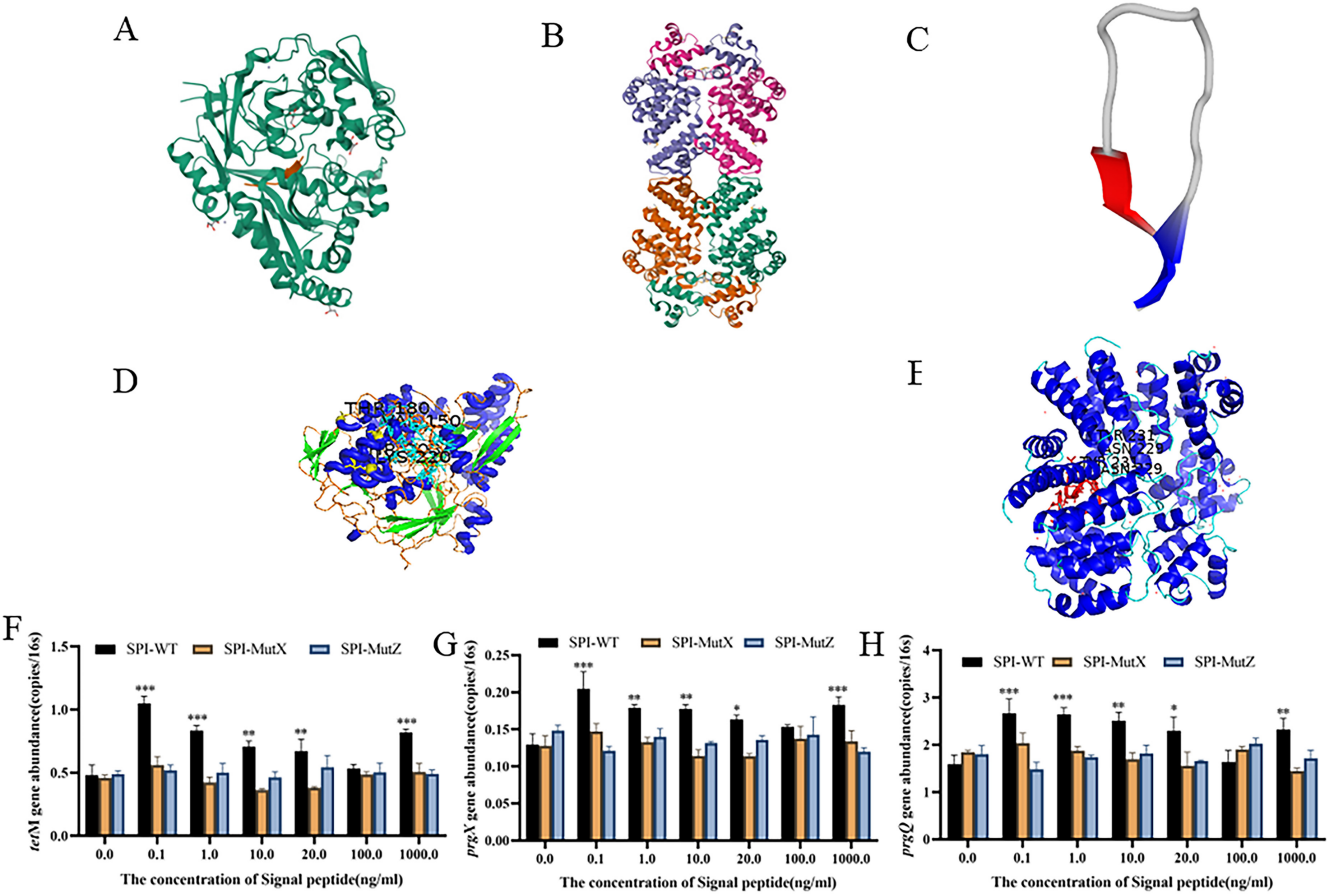
Structural, molecular docking, and docking-associated transfer experiments of the SPI-WT signal peptide from *Aerococcus urinaeequi* Ae1 with *prgX*, *prgZ*. **(A)** Three-dimensional structural model of *prgZ*; **(B)** Three-dimensional structural model of *prgX*; **(C)** Three-dimensional structural model of SPI-WT; **(D)** Molecular docking complex model of SPI-WT with *prgZ*; **(E)** Molecular docking complex model of SPI-WT with *prgX*. **(F–H)** Changes in the abundance of fusion-related genes under different concentrations of signal peptide treatment **(F)** *tetM*; **(G)** *prgX*; **(H)** *prgQ*. Significant differences were indicated as *P < 0.05, **P < 0.01, ***P < 0.001.

To further elucidate the mechanism of action of SPI-WT, this study first analyzed the tertiary structural features of *prgX* (DNA-binding domain), *prgZ* (pheromone-binding domain), and SPI-WT (Figures 6A–C), followed by molecular docking analysis. Subsequently, based on the key binding sites identified through docking screening, *prgX* binding site mutants (SPI-MutX) and *prgZ* binding site mutants (SPI-MutZ) were constructed (Figures 6D,E). The classical regulatory mechanism of conjugative transfer in *E. faecalis* has been well established: *prgZ* acts as a signal peptide transporter mediating intracellular signal peptide uptake (Kurnia et al., 2020), while *prgX* inhibits conjugation gene transcription by binding to the *prgQ* promoter region (Chatterjee et al., 2011). The peptide signal may target both proteins simultaneously to activate the regulatory pathway. Molecular docking results suggest that SPI-WT may bind to the pheromone-binding domain of *PrgZ* and the DNA-binding domain of *PrgX*, which is consistent with this classical regulatory mechanism. However, it should be noted that these docking results are merely computational predictions and still need to be validated through experiments, such as surface plasmon resonance or isothermal titration calorimetry. Based on this, we propose the hypothesis that SPI-WT may mimic the action mode of cCF10 by specifically binding to *PrgX* and *PrgZ* to achieve regulation, with the integrity of the binding sites being a prerequisite for its functional activity.

To validate this hypothesis, SPI-MutX and SPI-MutZ at different concentrations were added to the conjugation system for functional verification, with SPI-WT treatment groups serving as positive controls. Conjugation transfer results showed that the abundance of conjugation-related genes in SPI-MutX and SPI-MutZ treatment groups at all concentrations did not differ significantly from the control group (*P* > 0.05) and was significantly lower than that in the concurrent SPI-WT treatment group (Figures 6F–H). This result supports the hypothesis: SPI-WT, as an endogenous signal peptide of Ae1, may activate the intergeneric conjugation pathway by specifically binding to *prgX* and *prgZ*. The complete loss of its promoting function following binding site mutation further suggests that this signal peptide is the specific molecule involved in regulating intergeneric conjugation in Ae1.

### 3.7 Pheromone-like activity of SPI-WT

**FIGURE 7:**
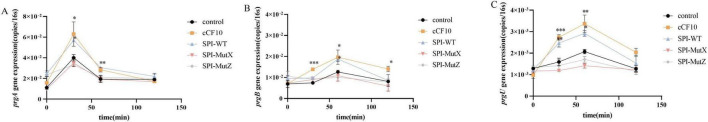
Pheromone effects of SPI-WT. Effects of SPI-WT on the expression of key regulatory genes involved in pCF10 conjugation. **(A)** *prgA*; **(B)** *prgB*; **(C)** *prgU*. Significant differences are indicated as *P < 0.05, **P < 0.01, ***P < 0.001.

The above results indicate that SPI-WT promotes the cross-species conjugative transfer of the pCF10 plasmid; however, whether it truly possesses pheromone-like activity requires further verification. To this end, we examined the effect of SPI-WT on the expression of key regulatory genes (*prgA*, *prgB*, *prgU*) in the pCF10 transfer system. The experimental results showed that in the cCF10-positive control group, prgA expression peaked at 30 min, while *prgB* and *prgU* peaked at 60 min, which is consistent with the known rapid induction characteristics of pheromones. Notably, the SPI-WT treatment group also exhibited expression peaks at 30 min (*prgA*) and 60 min (*prgB*, *prgU*), with an upregulation trend largely similar to that of the cCF10 group (Figures 7A–C). This phenomenon suggests that SPI-WT may exert transcriptional regulatory effects similar to those of cCF10, and their kinetics of action appear to be the same. Furthermore, we found that gene expression levels in the binding site mutant groups (SPI-MutX and SPI-MutZ) were similar to those in the control group at all time points, with no significant increase observed. This result suggests that the integrity of the *prgX* and *prgZ* binding sites may be a prerequisite for SPI-WT to exert its effects. In summary, although SPI-WT lacks sequence homology with cCF10, our data support the possibility that it may upregulate the expression of key conjugation-related genes by acting on PrgX and PrgZ. However, because direct evidence is lacking, its proposed pheromone-like activity remains hypothetical and awaits further experimental validation.

## 4 Discussion

Horizontal gene transfer, as the primary pathway for microorganisms to rapidly acquire ARGs, has emerged as a major challenge in global public health (Gabashvili et al., 2020). Studies indicate that environmental ARGs are highly likely to enter organisms via water and food sources (Silvester et al., 2025). Within these organisms, particularly in the gut, a vast reservoir of recipient microbial communities exists that is receptive to acquiring resistance genes, providing an ideal environment for their dissemination. Consequently, the gut has progressively become a “reservoir” for ARGs (Dhariwal et al., 2023). Given the gut’s role as a major reservoir for resistance genes, we hypothesize that within the complex intestinal microenvironment, certain poorly characterized bacterial strains exist that possess the capacity to mediate transfer of the pCF10 plasmid.

To validate this hypothesis, this study employed real-time quantitative PCR screening combined with dual-resistance plate verification to demonstrate that clinically isolated *Aerococcus urinaeequi* can serve as a recipient strain, acquiring the pCF10 plasmid from *E. faecalis*, most likely via a conjugation-like mechanism. This finding extends the conventional understanding that the host range of the pCF10 plasmid is primarily confined to the genus *Enterococcus* (Dunny, 2007; Kao et al., 1991). Regarding strain characteristics, the isolated strain shares similar clinical origins and physiological traits with previously reported *Aerococcus urinae* species. However, its key distinction lies in the ability to acquire the pCF10 plasmid, potentially stemming from genetic plasticity acquired under antibiotic selection pressure (Antiporta and Dunny, 2002). Regarding the potential mechanism of transfer, the observed transfer frequency (approximately 10^–3^) is lower than the highest efficiency within *E. faecalis* but remains significantly higher than the intergeneric transfer rates of most host plasmids. Crucially, the addition of exogenous cCF10 pheromone significantly enhances the transfer frequency–a phenomenon typically induced by endogenously secreted cCF10 in the *E. faecalis* conjugation system (Hirt et al., 2005). Further BLAST analysis confirmed the absence of cCF10-encoding genes in the Ae1 genome isolated in this study, indicating its inability to endogenously synthesize the cCF10 pheromone. This contrasts with the classical model where *E. faecalis* recipient strains rely on their own cCF10 for efficient conjugation induction (Antiporta and Dunny, 2002). This finding suggests that Ae1’s transfer potential must rely on exogenous cCF10 produced by donor bacteria for activation. This signal-dependent mode represents the key molecular mechanism enabling pCF10 plasmid’s cross-genus transfer, while also providing direct evidence for understanding the functional adaptation of pheromone regulatory systems in heterologous hosts (Hirt et al., 2005). In summary, this successful intergeneric transfer further indicates that the replication and maintenance system of the pCF10 plasmid is compatible with *Aerococcus urinaeequi*, suggesting that ARGs such as *tetM* carried by pCF10 may spread to more distantly related Gram-positive bacterial groups via opportunistic pathogens widely colonizing the gut and urogenital tract. We therefore propose that the observed pheromone-dependent, cell-contact-requiring transfer from *E. faecalis* to *Aerococcus urinaeequi* occurs via a conjugation-like mechanism. This significantly expands the transmission range of ARGs.

*Aerococcus urinae*, as clinically significant opportunistic pathogens initially isolated and identified from urine, are one of the common causative agents of urinary tract infections (UTIs) in middle-aged and elderly populations, particularly in complicated UTIs (Rast et al., 2023). With the widespread use of β-lactam, macrolide, and fluoroquinolone antibiotics in UTI treatment, the issue of uropathogens resistance has become increasingly prominent (Ramirez Castillo et al., 2023). Studies indicate that the resistance rate of *Aerococcus urinae* to commonly used clinical antibiotics has been increasing annually, with some strains exhibiting multidrug-resistant phenotypes, posing severe challenges to clinical treatment (Lamichhane et al., 2024). This study discovered that *Aerococcus urinaeequi* can acquire the pCF10 plasmid through cross-genus transfer, suggesting that this strain may accelerate the evolution of drug resistance by acquiring exogenous resistance plasmids. This finding carries significant implications for the prevention and control of clinically resistant infections. Of note, in this study *Aerococcus urinaeequi* was isolated from mouse gut microbiota, suggesting that this species may also colonize the intestinal tract. Given the gut’s role as a major reservoir of antibiotic resistance genes, the presence of *A. urinaeequi* in the intestine could further facilitate the acquisition and dissemination of resistance plasmids, thereby amplifying its clinical impact.

More importantly, this cross-genus transfer process may be regulated by pheromones. Sequence alignment revealed that the Ae1 genome lacks cCF10 homologous sequences yet responds to this signal and undergoes transfer, suggesting it may possess its own unique signal regulatory pathway. Further experiments suggested that Ae1’s unique secreted protein signal peptide SPI-WT may mediate this process. Our transcription activation assays showed that in the SPI-WT treatment group, *prgA* expression peaked at 30 min, while *prgB* and *prgU* peaked at 60 min, consistent with the trend observed in the cCF10-positive control group; in contrast, no significant upregulation of expression was observed in the binding site mutants SPI-MutX and SPI-MutZ. This expression kinetics is generally consistent with the pattern reported by Hirt et al. (2005), in which the overall transcriptional activity of pCF10 donor bacteria following cCF10 induction peaked between 30 and 60 min and subsequently declined after 2 h. It is known that the cCF10 phage inducer induces pCF10 donor cells to produce three surface adhesins (PrgA, PrgB, and PrgC), thereby activating the Type IV secretion system to facilitate efficient plasmid transfer (Bhatty et al., 2017). Among these, the surface protein Asc10, encoded by prgB, mediates aggregation between donor and recipient cells and is essential for high-frequency conjugative transfer (Olmsted et al., 1991); while prgU suppresses the cytotoxic effects of excessive PrgB following pheromone induction by downregulating PrgB synthesis, thereby maintaining cell membrane integrity and ensuring the normal progression of the conjugative transfer process (Bhatty et al., 2017). Since *prgA*, *prgB*, and *prgU* are downstream effector genes in the pCF10 conjugation regulation network, their upregulation suggests that upstream regulatory pathways may have been activated. Molecular docking results suggest that SPI-WT may bind to the pheromone-binding domain of PrgZ and the DNA-binding domain of PrgX, potentially regulating the mating pathway by relieving PrgX-mediated transcriptional repression and enhancing PrgZ transport efficiency. However, direct experimental evidence for such a mechanism–including physical binding, intracellular uptake, and relief of repression–is currently lacking. Nevertheless, mutations at these binding sites appear to eliminate this promoting effect, which is indirectly consistent with classical peptide signaling mechanisms (Breuer et al., 2018). Notably, SPI-WT’s primary function likely mediates extracellular transport of its associated secreted protein rather than specifically regulating pCF10 conjugation. Literature and database searches indicate SPI-WT is newly reported, while BLASTp analysis shows its core conserved fragment exists in the genus *Aerococcus* but lacks matches in *Enterococcus*, suggesting potential dissemination alongside secreted protein genes in closely related Gram-positive bacteria. This raises the possibility that such strains may secrete proteins containing homologous signal peptides, with conserved domains ensuring binding function, and coexist with Enterococcus in the gut microbiota. We acknowledge that although SPI-WT was identified as a putative signal peptide based on genomic prediction and shown to promote transfer in exogenous addition assays, we have not directly demonstrated that Ae1 naturally produces and secretes SPI-WT under physiological conditions. Therefore, the conclusion that “*Aerococcus urinae* secretes SPI-WT” remains hypothetical. Future studies using targeted proteomics on culture supernatants are required to confirm endogenous secretion and to determine its physiological concentration. This inference expands our understanding of the host range for pCF10 and suggests it may exacerbate the risk of antibiotic resistance gene spread.

In summary, this study provides initial evidence that *Aerococcus urinaeequi* mediates transgeneric transfer of the pCF10 plasmid, expanding the known host range of this plasmid while suggesting a novel pathway for transgeneric dissemination of intestinal ARGs. The combination of *Aerococcus urinaeequi*’s clinical pathogenicity and its ability to acquire antimicrobial resistance genes may exacerbate challenges in controlling drug-resistant infections. Its specific response to pheromone signals offers a novel perspective for elucidating the regulatory mechanisms of intergeneric conjugation in Gram-positive bacteria. While the transcriptional activation data and the loss-of-function effects of the binding-site mutants provide only indirect evidence, they consistently support a pheromone-like regulatory role of SPI-WT. Future studies should further clarify the molecular mechanisms by which *Aerococcus urinaeequi* recognizes pheromones, providing theoretical foundations and potential targets for developing strategies to block the intergeneric transmission of antibiotic resistance genes.

However, it should be acknowledged that this study has several limitations. First, although both bioinformatics predictions and exogenous peptide addition experiments suggest that SPI-WT plays a regulatory role, we have not yet directly demonstrated that *Aerococcus urinaeequi* can produce and secrete SPI-WT under physiological conditions. Second, molecular docking simulations predicted a potential interaction between SPI-WT and PrgX/PrgZ, but direct binding assays (such as surface plasmon resonance or isothermal titration calorimetry) have not yet been performed. Third, it remains unclear whether SPI-WT is taken up by donor cells and exerts its effects intracellularly–a requirement for the classical cCF10 signaling pathway. Fourth, although SPI-WT increases the transcriptional levels of conjugation-related genes, there is a lack of direct evidence (such as electrophoretic mobility shift assays) demonstrating that it can lift PrgX-mediated transcriptional repression. Fifth, the effect of SPI-WT on conjugation was assessed through qPCR-based gene abundance analysis rather than through direct quantification of transconjugant colony-forming units or mating aggregate formation. Therefore, the available data support an association between SPI-WT and enhanced conjugation, rather than a definitive causal relationship. These limitations highlight the need for future mechanistic studies, including targeted proteomics to detect endogenous SPI-WT secretion, fluorescence-based uptake assays, and direct transfer frequency measurements.

## 5 Conclusion

In this study, we provide the first experimental evidence that a strain of *Aerococcus urinaeequi* from the gut microbiota is capable of intergeneric transfer with *E. faecalis*. Based on our findings, we hypothesize that this *Aerococcus urinaeequi* strain may secrete an endogenous signal peptide (SPI-WT) that functions similarly to the pheromone cCF10 and may initiate gene transfer by regulating the prgX/prgZ operon in *E. faecalis*. However, direct evidence regarding the natural secretion, physical binding, cellular uptake, and transcriptional release of SPI-WT is currently lacking; therefore, this proposed mechanism remains a hypothesis requiring future validation. Despite these limitations, the discovery of the cross-genus transfer of pCF10 to *Aerococcus urinaeequi* provides new insights into the mechanisms of ARG transmission within the gut microbiota and highlights previously unrecognized molecular players in conjugative regulation.

## Data Availability

The 16S rRNA and whole genome shotgun sequencing data generated for this study have been deposited in GenBank under accession numbers PZ489770 (https://www.ncbi.nlm.nih.gov/nuccore/PZ489770) and JBYUIT000000000 (https://www.ncbi.nlm.nih.gov/nuccore/JBYUIT000000000.1).
