# Correction: A newly discovered *Aerococcus urinaeequi* mediates transfer of the pCF10 plasmid via SPI-WT regulation

**DOI:** 10.3389/fmicb.2026.1893541

**Published:** 2026-06-29

**Authors:** Man Zhang, Xiaobo Yang, Rumeng Li, Jingxue Qian, Ruolin Hao, Lin Xu, Qing He, Zhiqiang Shen, Jingfeng Wang, Yu Zhu, Zhigang Qiu

**Affiliations:** 1 The Third Central Clinical College of Tianjin Medical University, Tianjin, China; 2 Military Medical Sciences Academy, Academy of Military Sciences, Tianjin, China; 3 College of Oceanography and Ecological Science, Shanghai Ocean University, Shanghai, China; 4 School of Environmental and Chemical Engineering, Xi'an Polytechnic University, Xi'an, China; 5 Tianjin Key Laboratory of Extracorporeal Life Support for Critical Diseases, Department of Nutrition, Central Hospital, Artificial Cell Engineering Technology Research Center, Tianjin University/Tianjin Third Central Hospital, Tianjin, China; 6 Tianjin Institute of Geriatrics, Tianjin Third Central Hospital Branch, National Medical Quality Control Center of Clinical Nutrition, Tianjin, China

**Keywords:** *Aerococcus urinaeequi*, antibiotic resistance genes, cross-genus transfer, gut microbiota, pCF10 plasmid, signal peptide

In the published article, the species name of the bacterial isolate Ae1 was incorrectly given as *Aerococcus urinae*. Specifically, every instance of “*Aerococcus urinae*” (when referring to the isolate Ae1) has been replaced with “*Aerococcus urinaeequi*”. For clarity, the species name *Aerococcus urinae* in the cited literature (which refers to other strains, not isolate Ae1) is left unchanged. The clinical background discussion originally referencing *Aerococcus urinae* remains applicable to *Aerococcus urinaeequi*. Both species are opportunistic pathogens within the *Aerococcus urinae* complex, capable of causing comparable infections (including urinary tract infections) in humans, and exhibit similar phenotypic and antimicrobial resistance characteristics. In the Results section, the morphological, physiological, and biochemical comparisons of isolate Ae1 to previously reported *Aerococcus urinae* strains remain valid, because *Aerococcus urinaeequi* and *Aerococcus urinae* are indistinguishable by routine phenotypic tests. Therefore, only the species assignment of Ae1 has been corrected based on genomic data; all experimental results and biological conclusions are unaffected.

The title of this article was erroneously given as: A newly discovered *Aerococcus urinae* mediates transfer of the pCF10 plasmid via SPI-WT regulation

The correct title of the article is A newly discovered *Aerococcus urinaeequi* mediates transfer of the pCF10 plasmid via SPI-WT regulation.

The citation of this article was incorrectly given as: Zhang M, Yang X, Li R, Qian J, Hao R, Xu L, He Q, Shen Z, Wang J, Zhu Y and Qiu Z (2026) A newly discovered *Aerococcus urinae* mediates transfer of the pCF10 plasmid via SPI-WT regulation. *Front. Microbiol*. 17:1817926. doi: 10.3389/fmicb.2026.1817926

The correct citation of the article is: Zhang M, Yang X, Li R, Qian J, Hao R, Xu L, He Q, Shen Z, Wang J, Zhu Y and Qiu Z (2026) A newly discovered *Aerococcus urinaeequi* mediates transfer of the pCF10 plasmid via SPI-WT regulation. *Front. Microbiol*. 17:1817926. doi: 10.3389/fmicb.2026.1817926

The keywords were erroneously given as: *Aerococcus urinae*, antibiotic resistance genes, cross-genus transfer, gut microbiota, pCF10 plasmid, signal peptide

The corrected keywords are: *Aerococcus urinaeequi;* antibiotic resistance genes; cross-genus transfer; gut microbiota; pCF10 plasmid; signal peptide.

A correction has been made to the section **Abstract, Methods**, paragraph one, page 1:

The corrected paragraph appears below:

“This study isolated and screened a Gram-positive coccus, *Aerococcus urinaeequi* Ae1, from the gut microbiota, and confirmed that Ae1 can undergo intergeneric plasmid transfer with *Enterococcus faecalis* (*E. faecalis*), challenging the conventional understanding that the pCF10 plasmid spreads only within the same species.”

A correction has been made to the section **Abstract, Results and discussion**, paragraph one, page 1:

“This study suggests that *Aerococcus urinaeequi* can acquire the pCF10 plasmid via intergeneric transfer and provides preliminary evidence that its endogenous signal peptide SPI-WT may play a regulatory role via the *prgZ/prgX* pathway.”

A correction has been made to the Section **Introduction**, paragraph six, page 3:

“Through these experiments, a non-Enterococcus Grampositive bacterium– *Aerococcus urinaeequi* –was successfully isolated from the gut microbiota. This strain serves as a novel recipient for the pCF10 plasmid. This study experimentally demonstrates for the first time that the pCF10 plasmid can be transferred from *E. faecalis* to *Aerococcus urinaeequi*, revealing a previously unknown pathway for the cross-genus transmission of ARGs within the intestinal microenvironment.”

A correction has been made to the section **Results**, ***3.3 Morphological structure and*** ***physiological and biochemical indicator testing of Ae1***, paragraph two, page 8:

“Combined with prior morphological observations, this further confirms the strain as *Aerococcus urinaeequi*,.”

A correction has been made to the Section **Results**, ***3.4 Whole genome sequencing of*** ***isolate Ae1***, paragraph one, page 9:

“Further analysis using 120 conserved single-copy genes across bacteria, leveraging the GTDB database and GTDBtk classification tool, revealed that Ae1 exhibited the highest average nucleotide identity (ANI) of 97.74% with *Aerococcus urinaeequi* GCF_001543205.1. This ultimately identified the isolate as *Aerococcus urinaeequi*. To validate the classification, specific sequences from Ae1 were compared against NCBI database sequences. The top ten sequences with 100% coverage and >99% similarity were selected for homology analysis. After alignment using the ClustalW tool in MEGAX software, a phylogenetic tree was constructed via the Neighbor-Joining method (Figure 4D). Results indicated that the 16S rRNA sequence of Ae1 showed the highest homology with the standard strain TRG25 of *Aerococcus urinaeequi*, suggesting a strong genetic relationship between them.”

There was a mistake in the caption of Figure 4 as published. The corrected caption of Figure 4 appears below.

“Genomic sequencing characteristics of *Aerococcus urinaeequi*. Ae1 strain. **(A)** Circos plot of genome A; **(B)** Bar chart of gene length distribution; **(C)** Species annotation count statistics from NR database; **(D)** Phylogenetic tree based on *16S rRNA* gene sequences; **(E)** Functional classification diagram of PHI genes. **(A–C, E)** adapted from the bacterial genome framework project completion report of Shanghai Paisenuo Biotechnology Co., Ltd. (Project No.: PN20241120064); **(D)** generated based on completion report data and independent analysis results.”

There was a mistake in the caption of Figure 6 as published. The corrected caption of Figure 6 appears below.

“Structural, molecular docking, and docking-associated transfer experiments of the SPI-WT signal peptide from *Aerococcus urinaeequi*. Ae1 with *prgX, prgZ*. **(A)** Three-dimensional structural model of *prgZ*; **(B)** Three-dimensional structural model of *prgX*; **(C)** Three-dimensional structural model of SPI-WT; **(D)** Molecular docking complex model of SPI-WT with *prgZ*; **(E)** Molecular docking complex model of SPI-WT with *prgX*. **(F–H)** Changes in the abundance of fusion-related genes under different concentrations of signal peptide treatment **(F)** *tetM*; **(G)** *prgX*; **(H)** *prgQ*. Significant differences were indicated as ^*^P < 0.05, ^**^P < 0.01, ^***^P < 0.001.”

A correction has been made to the Section **Discussion**, paragraph two, page 12:

“To validate this hypothesis, this study employed real-time quantitative PCR screening combined with dual-resistance plate verification to demonstrate that clinically isolated *Aerococcus urinaeequi*. can serve as a recipient strain, acquiring the pCF10 plasmid from *E. faecalis*, most likely via a conjugation-like mechanism.”

A correction has been made to the Section **Discussion**, paragraph two, page 13:

“In summary, this successful intergeneric transfer further indicates that the replication and maintenance system of the pCF10 plasmid is compatible with *Aerococcus urinaeequi*, suggesting that ARGs such as tetM carried by pCF10 may spread to more distantly related Gram-positive bacterial groups via opportunistic pathogens widely colonizing the gut and urogenital tract. We therefore propose that the observed pheromone-dependent, cell-contact-requiring transfer from *E. faecalis* to *Aerococcus urinaeequi* occurs via a conjugation-like mechanism. This significantly expands the transmission range of ARGs.”

A correction has been made to the Section **Discussion**, paragraph three, page 13:

“This study discovered that *Aerococcus urinaeequi* can acquire the pCF10 plasmid through cross-genus transfer, suggesting that this strain may accelerate the evolution of drug resistance by acquiring exogenous resistance plasmids. This finding carries significant implications for the prevention and control of clinically resistant infections. Of note, in this study *Aerococcus urinaeequi* was isolated from mouse gut microbiota, suggesting that this species may also colonize the intestinal tract. Given the gut's role as a major reservoir of antibiotic resistance genes, the presence of *A. urinaeequi* in the intestine could further facilitate the acquisition and dissemination of resistance plasmids, thereby amplifying its clinical impact.”

A correction has been made to the Section **Discussion**, paragraph four, page 13:

“Therefore, the conclusion that “*Aerococcus urinaeequi* secretes SPI-WT” remains hypothetical.”

A correction has been made to the Section **Discussion**, paragraph five, page 13:

“In summary, this study provides initial evidence that *Aerococcus urinaeequi* mediates transgeneric transfer of the pCF10 plasmid, expanding the known host range of this plasmid while suggesting a novel pathway for transgeneric dissemination of intestinal ARGs. The combination of *Aerococcus urinaeequi*'s clinical pathogenicity and its ability to acquire antimicrobial resistance genes may exacerbate challenges in controlling drug-resistant infections.”

A correction has been made to the Section **Discussion**, paragraph five, page 14:

“Future studies should further clarify the molecular mechanisms by which *Aerococcus urinaeequi* recognizes pheromones, providing theoretical foundations and potential targets for developing strategies to block the intergeneric transmission of antibiotic resistance genes.”

A correction has been made to the Section **Discussion**, paragraph six, page 14:

“First, although both bioinformatics predictions and exogenous peptide addition experiments suggest that SPI-WT plays a regulatory role, we have not yet directly demonstrated that *Aerococcus urinaeequi* can produce and secrete SPI-WT under physiological conditions.”

A correction has been made to the Section **Conclusion**, paragraph one, page 14:

“In this study, we provide the first experimental evidence that a strain of *Aerococcus urinaeequi* from the gut microbiota is capable of intergeneric transfer with *E. faecalis*. Based on our findings, we hypothesize that this *Aerococcus urinaeequi* strain may secrete an endogenous signal peptide (SPI-WT) that functions similarly to the pheromone cCF10 and may initiate gene transfer by regulating the prgX/prgZ operon in *E. faecalis*. However, direct evidence regarding the natural secretion, physical binding, cellular uptake, and transcriptional release of SPI-WT is currently lacking; therefore, this proposed mechanism remains a hypothesis requiring future validation. Despite these limitations, the discovery of the cross-genus transfer of pCF10 to *Aerococcus urinaeequi* provides new insights into the mechanisms of ARG transmission within the gut microbiota and highlights previously unrecognized molecular players in conjugative regulation.”

The **Data availability statement** was erroneously given as:

“The datasets presented in this study can be found in online repositories. The names of the repository/repositories and accession number(s) can be found below: https://db.cngb.org/ search/project/CNP0008936/, CNP0008936.”

The correct **Data availability statement** is:

“The 16S rRNA and whole genome shotgun sequencing data generated for this study have been deposited in GenBank under accession numbers PZ489770 (https://www.ncbi.nlm.nih.gov/nuccore/PZ489770) and JBYUIT000000000 (https://www.ncbi.nlm.nih.gov/nuccore/JBYUIT000000000.1).”

The original version of this article has been updated.

