# Supplementary material for: A newly discovered Aerococcus urinaeequi mediates transfer of the pCF10 plasmid via SPI-WT regulation
Source: Front Microbiol. 2026 May 20;17:1817926. doi: 10.3389/fmicb.2026.1817926 (PMC13230114; doi:10.3389/fmicb.2026.1817926)
Supplement: Supplementary file 1 [file Supplementary_file_1.docx]

# *A newly discovered Aerococcus urinae mediates transfer of the pCF10 plasmid via SPI-WT regulation*

**Man Zhang^1, 2,†^,Xiaobo Yang^2, †^, Rumeng Li ^2,3^ , Jingxue Qian ^2,4^, Ruolin Hao ^2^ , Lin Xu ^2,3^, Qing He^2^, Zhiqiang Shen ^2^ , Jingfeng Wang ^2^, Yu Zhu^1,^ ^5,6^ ^*^, Zhigang Qiu ^2, *^**

^1^ The Third Central Clinical College of Tianjin Medical University, Tianjin 300170, China

^2^ Military Medical Sciences Academy, Academy of Military Sciences, Tianjin 300050, China

^3^ College of Oceanography and Ecological Science, Shanghai Ocean University, Shanghai 201306, China

^4^ School of Environmental and Chemical Engineering, Xi’an Polytechnic University, Xian 710699, China

^5^ Tianjin Key Laboratory of Extracorporeal Life Support for Critical Diseases, Department of Nutrition, Central Hospital, Artificial Cell Engineering Technology Research Center, Tianjin University/Tianjin Third Central Hospital, Tianjin 300170, China

^6^ Tianjin Institute of Geriatrics, Tianjin Third Central Hospital Branch, National Medical Quality Control Center of Clinical Nutrition, Tianjin 300170, China

^†^These authors have contributed equally to this work and share first authorship.

*** Corresponding authors:**

E-mail addresses: zhuyutj@126.com (Y. Zhu), zhigangqiu99@gmail.com (Z. Qiu).

# Supplementary Texts

**Text S1** **Verification of the presence of OG1RF (pCF10) related genes in the genome of Ae1**

The genomic DNA of *Aerococcus urinae* was extracted using the bacterial genomic DNA extraction kit. At the same time, specific primers for the prgQ, prgX, ccfA and prgZ genes on the pCF10 plasmid were selected for PCR experiments and gel electrophoresis was performed to verify the PCR products.

**Text S2 Induction of Ae1 containing streptomycin resistance**

Cultivate Ae1 in BHI liquid medium until the logarithmic phase, then add streptomycin to a final concentration of 1 g/L. Continue culturing for 8 hours before transferring to fresh BHI liquid medium containing 1 g/L streptomycin. After 8 h of incubation, take 100 μL of the culture and spread it onto BHI agar medium containing 1 g/L streptomycin. Incubate at 37°C for 12 h, then pick out individual colonies for further culture. Repeat the above steps after increasing the streptomycin concentration to 2 g/L and 3 g/L, ultimately inducing streptomycin-resistant Ae1.

**Text S3 plate spread experiment to Verify Transfer**

To directly verify whether the pCF10 plasmid had been transferred from the donor strain to the recipient strain Ae1, we performed a plate spread experiment. The donor strain was E. faecalis OG1RF(pCF10) (tetracycline-resistant), which carried the pCF10 plasmid, and the recipient strain was *Aerococcus urinae* Ae1 (non-tetracycline-resistant). The donor and recipient bacteria were cultured overnight separately, and the bacterial suspensions were adjusted to an OD600 of approximately 0.5 (approximately 1 × 10⁸ CFU/mL). Equal volumes of the donor and recipient bacteria were mixed to form the transfer mixture; a separate culture of the donor bacteria was set up as a control. The transfer mixture and the separate donor culture were incubated at 37°C for 2 hours. After incubation, both sets of bacterial suspensions were serially diluted 10-fold. For each dilution (10⁻³, 10⁻⁴, 10⁻⁵), 100 μL was spread onto BHI plates containing tetracycline (10 mg/L). The 10⁻⁵ dilution was specifically used for colony counting to quantify the number of zygotes. Additionally, the isolated recipient strain Ae1 was plated separately on a tetracycline-containing plate as a negative control. After incubating all plates upside down at 37°C for 24 hours, the colonies were observed and counted.

**Text S4 Ae1 Physiological and Biochemical Indicator Testing**

Enzyme Assay: Streak inoculate test strain Ae1 and Enterococcus faecalis OG1RF (pCF10) separately onto TSA agar plates. incubate at 37°C for 18-24 hours to obtain fresh single colonies. Place one drop of freshly prepared 3% H₂O₂ solution onto a sterile microscope slide. Using a sterile inoculating loop, pick a single colony and place it into the solution, then grind and mix thoroughly. Observe within 10 seconds whether bubbles form. The presence of bubbles indicates a positive result, while the absence of bubbles indicates a negative result.

V-P Test: Inoculate Ae1 and Enterococcus faecalis OG1RF (pCF10) separately into sterile tubes containing 3 mL V-P medium. Incubate statically at 37°C for 48 hours. Transfer 2 mL of the culture into a new tube. First add 0.6 mL of 400 g/L α-naphthol ethanol solution and shake well. followed by 0.2 mL of 400 g/L potassium hydroxide solution containing 0.3% creatine. Incubate at 37°C for 15-30 min. Observe for red or pink color development; positive result indicates color change, negative result indicates no color change.

6.5% NaCl Growth Test: Inoculate Ae1 and OG1RF (pCF10) onto freshly prepared 6.5% NaCl medium. Incubate at 37°C for 24-48 hours. Observe whether the medium becomes turbid. Turbidity with color change indicates a positive result; clear medium with no color change indicates a negative result.

Cholestanol Test: Using a sterile inoculating loop, transfer fresh pure colonies of Ae1 and OG1RF (pCF10) onto cholestanol agar plates. Incubate at 37°C for 24–48 hours. Observe for black precipitation; presence indicates positive, absence indicates negative.

Arabinose and Trehalose Fermentation Test: Inoculate Ae1 and OG1RF (pCF10) separately into arabinose and trehalose fermentation tubes. Incubate at 37°C for 24-48 hours. Observe changes in medium color: a shift from purple to yellow indicates positive acid production via fermentation; retention of purple color indicates negative.

# Supplementary Figures and Tables

## Supplementary Figures


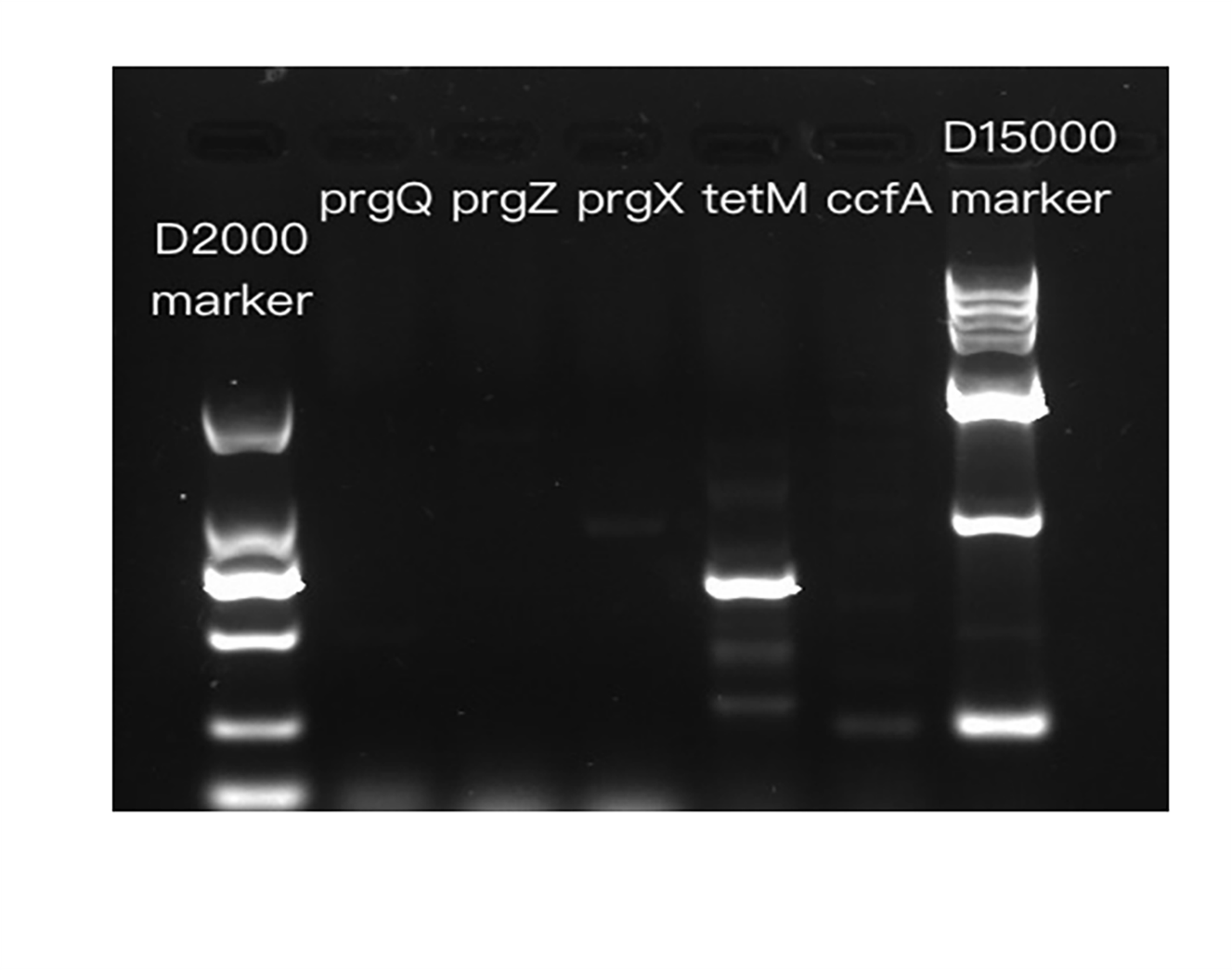


**Supplementary Figure S1.** Electrophoretic pattern of PCR products from the genome of *Ae1*.


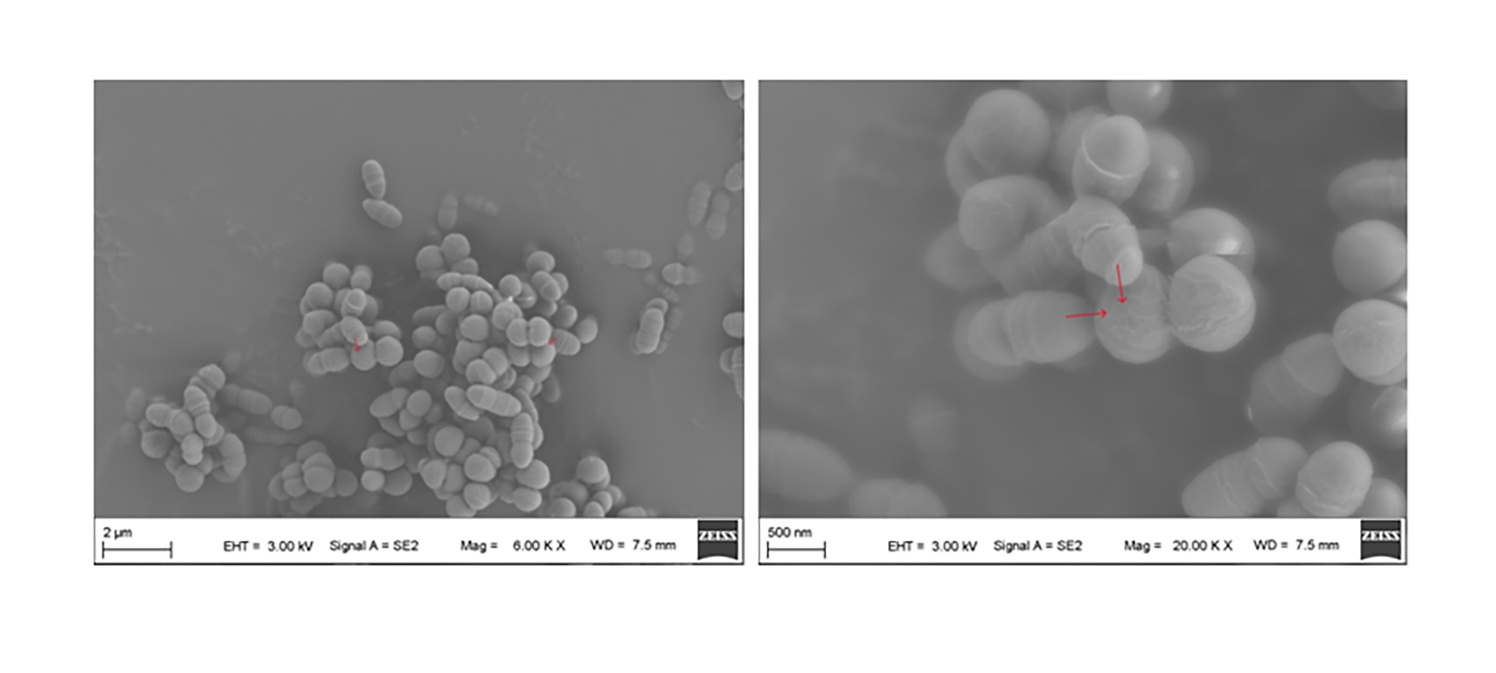


**Supplementary Figure S2.** Observation of *Ae1* and bacteria in the OG1RF (pCF10) conjugation system using an electron microscope (Left image magnified 6.00 KX, right image magnified 20.00 KX).

**
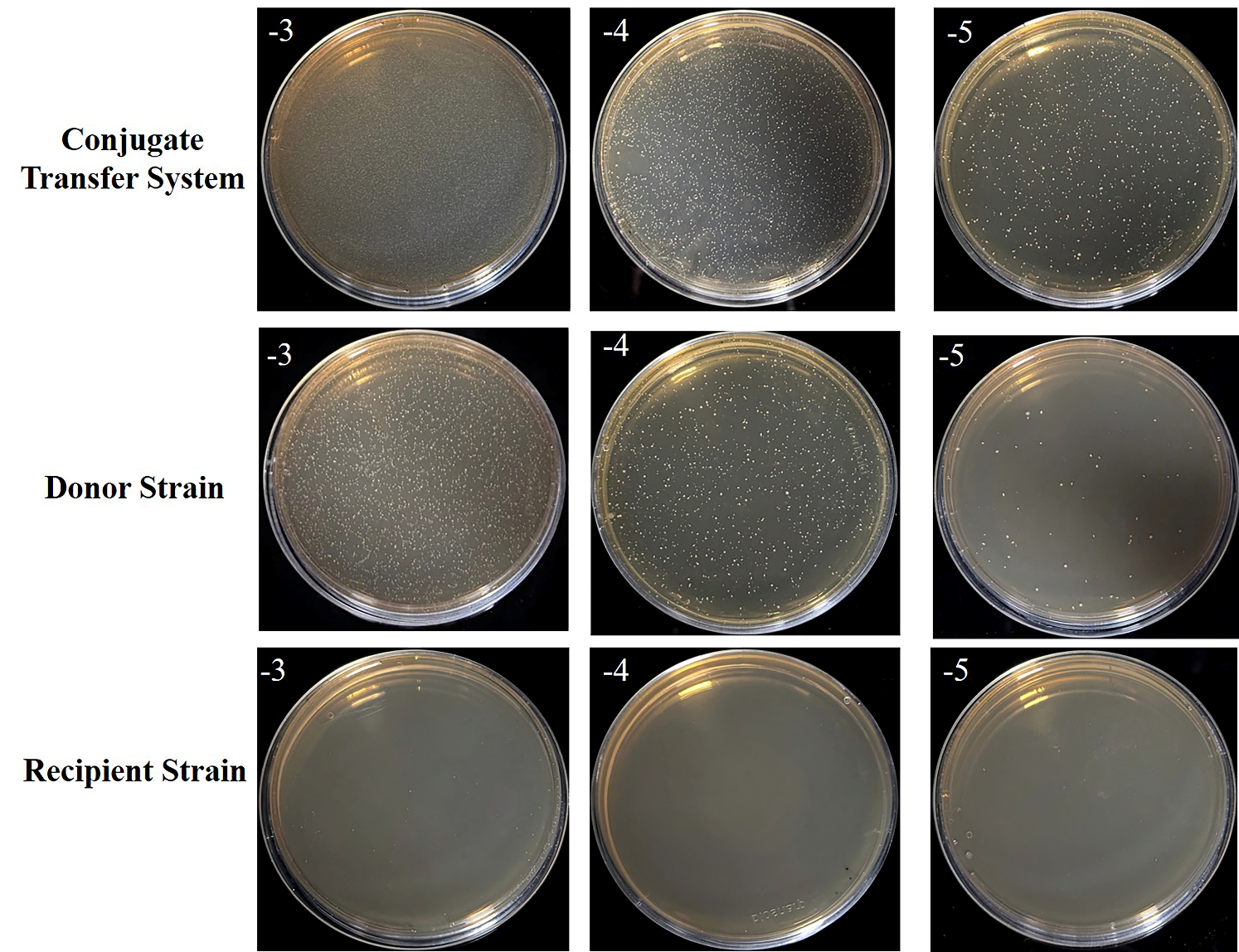
Supplementary Figure S3.** Droplet assay confirming the transfer of the pCF10 plasmid from the donor bacterium to Ae1


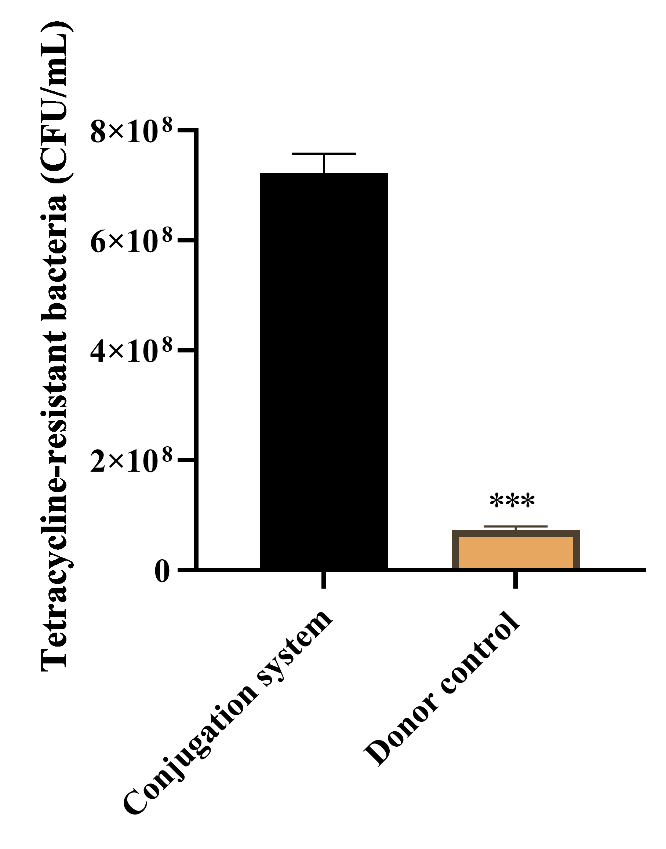


**Supplementary Figure S4.** Concentration of tetracycline-resistant bacteria in the donor-recipient system (CFU/mL)


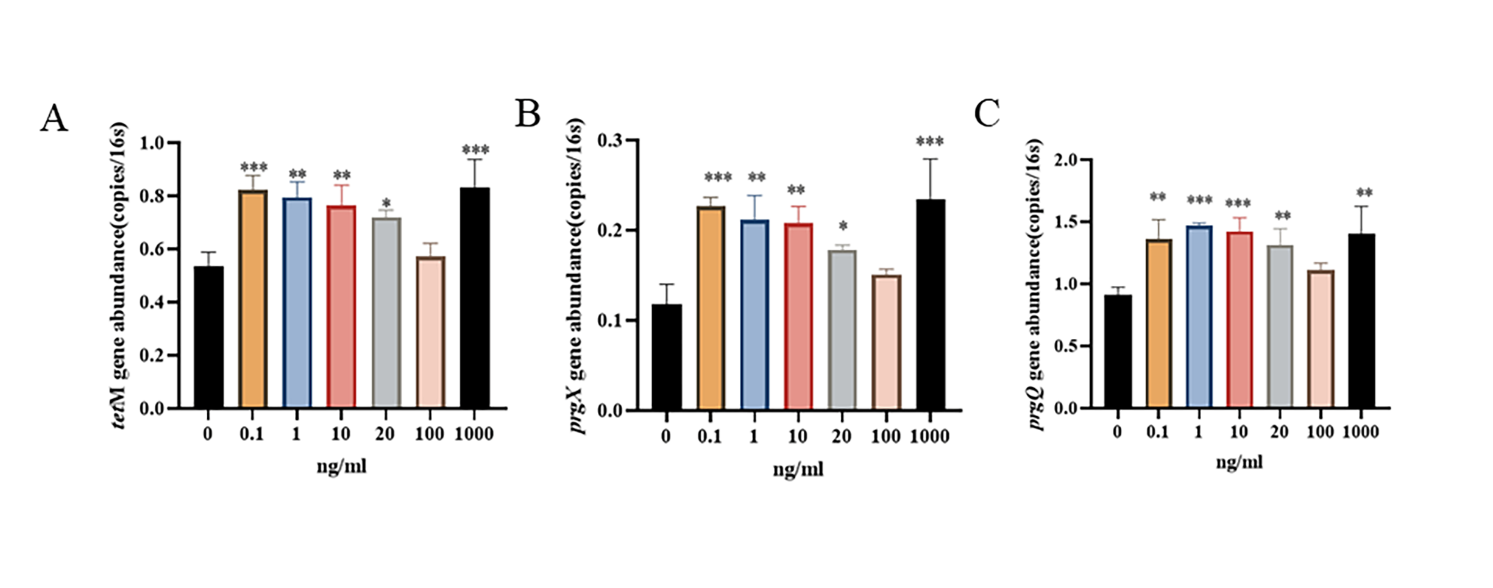


**Supplementary Figure S5.** Experiment on the Effect of SPI-WT on transfer. The significance of the differences was indicated by **P < 0.05, **P < 0.01, ***P < 0.001.*

## Supplementary Tables

**Table S1. List of bacteria and plasmids.**

| Strains or plasmids | Description | |
| --- | --- | --- |
| pCF10 | pheromone-responsive plasmid, Tet^R^ | |
| OG1RF(pCF10) | *E. faecalis* ATCC 47077 containing pCF10 plasmid, Tet^R^, Rif^R^ | |
| Ae1 | Fecal-isolated strains |  |
| Ae1-S | Fecal-isolated strains ,Str^R^ | |

**Table S2. Sequence of primers for qRT-PCR analysis**

| Gene name | Primer Sequence (5’ to 3’) | | Length of the product(bp) | |  |
| --- | --- | --- | --- | --- | --- |
| *ccfA* | F: AGATATACGAAGGCCCGCTGAA | | 243 | |  |
|  | R: AATTAGTTATCCGCTGCTCCTGA | |  |  |  |
| *prgQ* | F: TATAGGAGGGGTGTAAATGAAAAC | | 143 | |  |
|  | R: CCGTCCTATGGCAGTCG | |  |  |  |
| *prgX* | F: AGTTTTTCTTTTCCTGTTTCATTT  R: GACTCTCGACCGATTTCTGTAG | | 125 | |  |
| *tetM* | F:GATTTGGCGGCACTT | | 294 | |  |
|  | R：CGCACCCTCTACTACAAA | |  |  |  |
| *prgZ* | F: GACTTTGACTGCAGGGACACC | | 202 | |  |
|  | R: AGCGCCATCTCTAATCACAAT | |  |  |  |
| *16S rRNA* | | 16S F: CGGTGAATACGTTCYCGG | | 123 | |
|  |  | 16S R: GGWTACCTTGTTACGACTT | |  |  |
| *prgA* | | F:GTTTGATGATGGCCGCTTAGGTT | | 175 | |
|  | | R:CACTTCGCCGTTTTGGTATTGACT | |  |  |
| *prgB* | | F:AGCGGATGGAAAATTTTACTCACC  R:GCGCACTAGATACAGGCACATTA | | 197 | |
| *prgU* | | F: GCAGAAAGAGAGGCAAAGGG  R: CCACATTTCCATTGCACGTGT | | 129 | |

**Table S3. Antibiotic sensitivity test results**

| Serial number | antibiotic | Antimicrobial susceptibility |
| --- | --- | --- |
| 1 | Streptomycin | R |
| 2 | Tetracycline | R |
| 3 | Chloramphenicol | S |
| 4 | Cefotaxime | S |
| 5 | Polymyxin B | S |
| 6 | Vancomycin | R |
| 7 | Ofloxacin | S |
| 8 | Fosfomycin | S |
| 9 | Penicillin | R |
| 10 | Novobiocin | R |
| 11 | Erythromycin | S |
| 12 | Cefepime | S |
| 13 | Cefoperazone/Sulbactam | R |
| 14 | Water (negative control) | R |

**Table S4. PCR reaction system**

| Reagent | Volume (uL) |
| --- | --- |
| Taq Mix (2×) | 12 |
| ddH_2_O | 9 |
| Forward primer (10 umoL/L) | 1 |
| Reverse primer(10 umoL/L) | 1 |
| DNA template | 2 |
| Total volume | 25 |
